# Preserved local but disrupted contextual figure-ground influences in an individual with abnormal function of intermediate visual areas

**DOI:** 10.1016/j.neuropsychologia.2012.02.024

**Published:** 2012-06

**Authors:** Joseph L. Brooks, Sharon Gilaie-Dotan, Geraint Rees, Shlomo Bentin, Jon Driver

**Affiliations:** a UCL Institute of Cognitive Neuroscience, University College London, 17 Queen Square, London WC1N 3AR, UK; b Wellcome Trust Centre for Neuroimaging, University College London, 12 Queen Square, London WC1N 3BG, UK; c Department of Psychology & Center for Neural Computation, Hebrew University of Jerusalem, Jerusalem 91905, Israel

**Keywords:** Figure-ground organization, Segmentation, Perceptual organization, Context, Gestalt, Developmental visual agnosia, Grouping, Integration

## Abstract

Visual perception depends not only on local stimulus features but also on their relationship to the surrounding stimulus context, as evident in both local and contextual influences on figure-ground segmentation. Intermediate visual areas may play a role in such contextual influences, as we tested here by examining LG, a rare case of developmental visual agnosia. LG has no evident abnormality of brain structure and functional neuroimaging showed relatively normal V1 function, but his intermediate visual areas (V2/V3) function abnormally. We found that contextual influences on figure-ground organization were selectively disrupted in LG, while local sources of figure-ground influences were preserved. Effects of object knowledge and familiarity on figure-ground organization were also significantly diminished. Our results suggest that the mechanisms mediating contextual and familiarity influences on figure-ground organization are dissociable from those mediating local influences on figure-ground assignment. The disruption of contextual processing in intermediate visual areas may play a role in the substantial object recognition difficulties experienced by LG.

## Highlights

► We tested a developmental agnosic patient, LG. ► LG shows preserved local but impaired contextual influences on figure-ground. ► LG shows impaired familiarity influences on figure-ground. ► Visual areas V2/V3 play a role in figure-ground context and familiarity effects. ► Local, contextual, and familiarity figure-ground influences are dissociable.

## 1 Introduction

In the normally developed human visual system, visual scenes are effortlessly and rapidly segmented into a structured set of surfaces and objects. Yet it remains far from clear how the visual system achieves such a complex computation. Gestalt psychologists first pointed out that visual perception does not comprise only perception of independent local elements (Wertheimer, 1923). Rather, perception of local elements can depend on their surrounding context (for recent examples see: Anderson, 2003; Gilchrist, 1977; Herzog, 2003; Polat & Sagi, 1993; Rock & Palmer, 1990; Williams, McCoy, & Purves, 1998). There has been substantial interest in studying the mechanisms of such contextual integration (e.g. Albright & Stoner, 2002). A recurring issue is the level of visual processing at which various contextual influences first arise (e.g. Palmer, Brooks, & Nelson, 2003; Palmer & Nelson, 2000; Rock & Brosgole, 1964; Rock, Nijhawan, Palmer, & Tudor, 1992; Schulz & Sanocki, 2003) and its neural underpinnings. Neurophysiological findings shed some light on this. For instance, recent work suggests that perceived brightness (as induced by surrounding context) may be reflected in the activity of neurons as early as primary visual cortex (e.g. Harris, Schwarzkopf, Song, Bahrami, & Rees, 2011; MacEvoy, Kim, & Paradiso, 1998; Rossi & Paradiso, 1999). Other contextual influences may involve the input of higher-order visual areas with larger receptive fields. For instance, certain Gestalt grouping processes may involve not only horizontal but also feedback connections (e.g. Grossberg, Mingolla, & Ross, 1997; Roelfsema, 2006) from higher areas. This notion is supported by functional imaging and neurophysiological data (e.g. Fang, Kersten, & Murray, 2008; Lee & Nguyen, 2001).

**Fig. 1:**
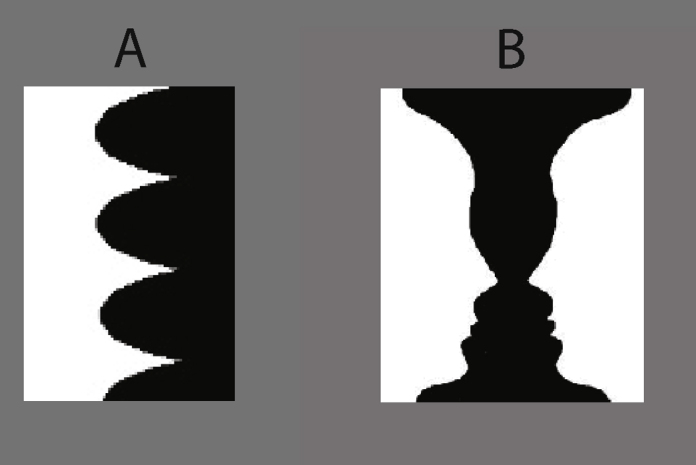
Examples of how perceived shape depends on figure-ground organization. (A) The shape along the central vertical edge can be perceived as a white object with sharp, spiked points on a black background or a black object with soft, rounded bumps on a white background. Which of these two interpretations is perceived depends on figure-ground organization across the edge. (B) The well-known faces-vase drawing by Rubin demonstrate how the same edges can depict either two profile faces or a central vase depending on the figure-ground organization across the edges.

Recent work shows that figure-ground assignment, a well-known topic in Gestalt psychology, can be determined by remote contextual visual information, in addition to the many well-known local figure-ground cues (Brooks & Driver, 2010; Peterson & Salvagio, 2008; Zhang & von Der Heydt, 2010). Figure-ground assignment is an important aspect of visual processing whereby contours are assigned to one or the other adjacent regions to determine relative depth and perceived shape along edges (e.g. Baylis & Driver, 2001; Burge, Peterson, & Palmer, 2005; Driver & Baylis, 1996; Nakayama, Shimojo, & Silverman, 1989). For instance, the visual system could interpret the edge in Fig. 1A as a black object with soft, rounded bumps (on a white background) which may feel nice to touch or, alternatively, as a white object with sharp, spiked points that should be avoided. Such figure-ground assignment ambiguity has often been demonstrated with the well-known faces-vase drawing (Fig. 1B) described by Rubin (1921). Figure-ground assignment is influenced by local image factors such as convexity (Kanizsa & Gerbino, 1976; Stevens & Brookes, 1988), edge-region relationships (Palmer & Brooks, 2008), surface curvature information (Palmer & Ghose, 2008), as well as cognitive factors such as previous experience (Peterson & Enns, 2005; Peterson & Gibson, 1994a; Peterson, Harvey, & Weidenbacher, 1991) and attention (Baylis & Driver, 1995; Huang & Pashler, 2009; Vecera, Flevaris, & Filapek, 2004). Neurons in V1 and V2 code the direction of figure-ground assignment across an edge (Fang, Boyaci, & Kersten, 2009; Qiu & von Der Heydt, 2005; Zhou, Friedman, & von Der Heydt, 2000) suggesting that early visual areas can have a strong representation of this important visual property. However, responses of these neurons are notably modulated by contextual information that falls outside the neuron's classical receptive field (Zhang & von Der Heydt, 2010). This suggests a mechanism which must integrate information across the visual field through connections with other neurons perhaps including feedback from higher level neurons with larger receptive fields (Jehee, Lamme, & Roelfsema, 2007; Roelfsema, Lamme, Spekreijse, & Bosch, 2002).

Neurophysiological work in V1 and V2 neurons (Sugihara, Qiu, & von der Heydt, 2011) has compared the onset latency of contextually driven figure-ground effects to that of locally-induced figure-ground effects and found that contextual influences are likely to involve feedback from higher-order visual areas. Some computational models (Craft, Schütze, Niebur, & von der Heydt, 2007; Jehee et al., 2007; Kienker, Sejnowski, Hinton, & Schumacher, 1986; Roelfsema et al., 2002; Sajda & Finkel, 1995) also support this notion, while others suggest that figure-ground computations are confined to early visual cortex (e.g. Baek & Sajda, 2005; Nishimura & Sakai, 2004, 2005; Zhaoping, 2005). At present, it is unclear whether some of the recently established contextual influences on figure-ground assignment (e.g. Brooks & Driver, 2010; Peterson & Salvagio, 2008) reflect the same mechanism as local figure-ground cues, or whether they may be dissociable.

Here, we sought to address whether local and contextual mechanisms of figure-ground assignment are dissociable by studying a rare case of developmental visual agnosia, case LG (Aviezer, Hassin, & Bentin, 2011; Gilaie-Dotan, Bentin, Harel, Rees, & Saygin, 2011; Gilaie-Dotan, Perry, Bonneh, Malach, & Bentin, 2009). LG has no gross structural brain abnormality visible on MRI. Functional MRI experiments, however, show that while his primary visual cortex is functionally preserved his intermediate visual regions (V2/V3, see Gilaie-Dotan et al., 2009), both ventrally and dorsally, show functional pathology (e.g. are significantly deactivated in response to visual stimulation). Likewise ERP experiments also confirm that components normally associated with processing in intermediate visual cortices are abnormal or absent in LG (Gilaie-Dotan et al., 2009). This rare case, with preserved early visual function but pathological intermediate visual cortical function, provides an opportunity to test whether contextual influences on figure-ground assignment depend on normal functioning of intermediate visual areas, whereas local influences on figure-ground assignment may be computed in earlier visual cortex without feedback from higher level visual areas. If so, then local figure-ground assignment should be preserved in LG, whereas contextual influences should be reduced or eliminated. In addition, since LG also shows object recognition problems, we were further able to test whether top-down object-knowledge nonetheless influences his figure-ground assignment, as in normal individuals (e.g. Peterson & Gibson, 1991, 1994a).

## 2 Case history

LG was a 24 year old, right-handed male at the time of testing who suffers from developmental visual agnosia. His object and face recognition problems were already apparent from a very young age and he was formally diagnosed at the age of 8 years (Ariel & Sadeh, 1996). As part of a previous study (Gilaie-Dotan et al., 2009), a high-resolution structural MRI scan of his brain was examined by a neuro-radiologist who was unaware of LG's condition. No evidence of structural abnormality was identified. LG functions as a fully independent adult, studies, works and reads, after finishing high school successfully.

A brief overview of the object recognition neuropsychological examination that LG underwent is provided here. More details can be found in an earlier report (Gilaie-Dotan et al., 2009). In the Boston Naming Test, he scored within normal range (45/54, all failures explained by cultural factors) but in the Hooper Visual Organization Test, when objects are presented as a collection of their spatially scattered parts so that their identification requires re-orienting and integrating the parts into a whole, his performance was very weak (12.5/30, categorized as “very high probability of impairment”). While his performance with the minimal feature match, BORB-7 (Riddoch & Humphreys, 1993) was errorless, his ability to recognize overlapping line drawings, BORB-6; (Riddoch & Humphreys, 1993), was deficient. He performed better with simple geometrical shapes and had a noticeable difficulty with letters and more complex line drawings. This difficulty was reflected both by errors (e.g. 11 errors out of 36 superimposed triples of letters) and particularly by extremely long RTs. The ratio between the RTs of overlapping stimuli compared with RTs of isolated stimuli was three times the ratio of the normal mean. He is also severely prosopagnosic as assessed by the Benton Facial Recognition Test (Benton, Sivan, Hamsher, Varney, & Spreen, 1983): 33/54 faces, severely impaired; Warrington's Face/Word Recognition Test (Warrington, 1984), words = 47/50, faces = 37/50; and the Cambridge Face Memory Test (Duchaine & Nakayama, 2006), 34/75, 6 points below the usual congenital prosopagnosia mean.

Testing LG's brain activation patterns to visual stimulation as measured by fMRI (Gilaie-Dotan et al., 2009) revealed that activity in LG's primary visual cortex is apparently normal whereas LG's intermediate visual regions (corresponding to V2 and probably V3/VP) are profoundly deactivated by visual stimulation. This deactivation is independent of the precise nature of the visual stimuli (line drawings, gray scale or color photographs and even movie clips) and of the task performed (naming, 1-back, fixation, or free viewing). Interestingly, despite these profound and abnormal deactivations of intermediate visual cortex in response to visual stimulation, LG's higher-level visual regions are activated above baseline although object and face selectivity is impaired. LG's abnormal activation patterns were further confirmed in a series of ERP experiments. The visual N1 component, associated with neural activity in intermediate visual regions, was abnormal, and the face-selective N170 component did not differ in amplitude between faces and objects as it does in normal individuals.

## 3 Experiment 1

**Fig. 2:**
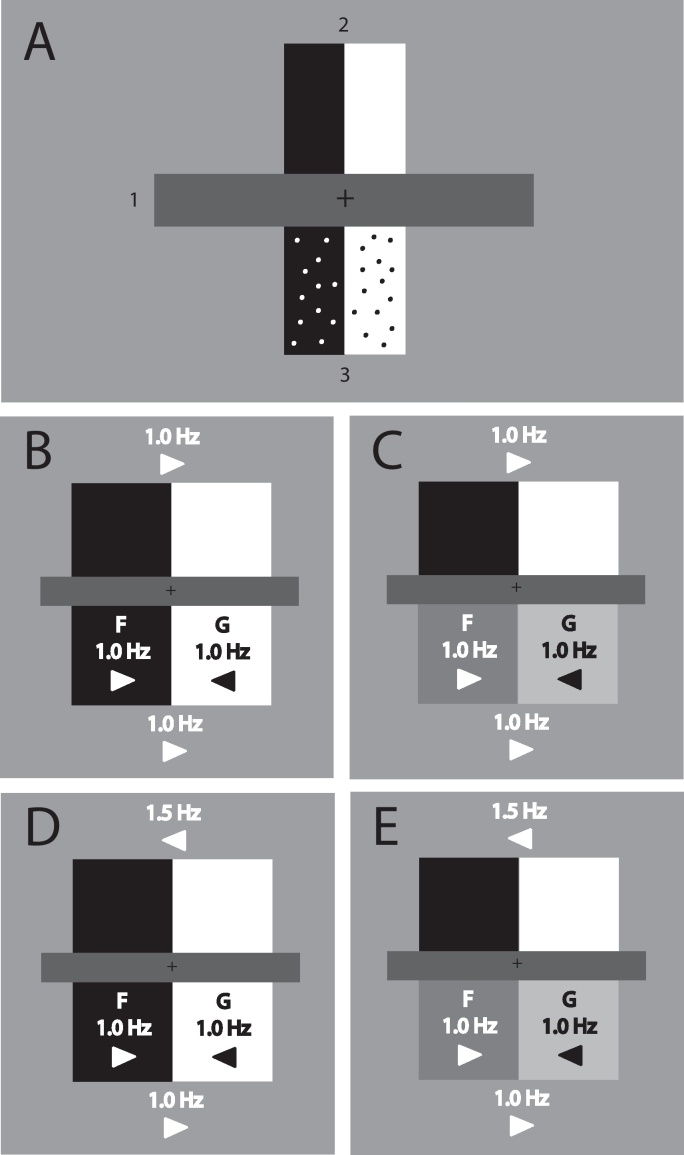
Displays used in Experiment 1. (A) A static scale version of the stimulus. Each display comprised a red central horizontally oriented bar (gray here and labeled *3*) with superimposed fixation cross and one bipartite section above and one bipartite section below it. Each bipartite section contained a vertically oriented dividing edge (luminance contrast) which we call the *critical edge* (labeled *2* and *3* here for each section separately). In this example, the locally-ambiguous section is located above the red (gray here) bar (and its critical edge is labeled *2*) and the lower section is the locally-biased section in this case (and its critical edge is labeled *3*). The location of the locally-ambiguous and locally-biased sections was counterbalanced in Experiment 1. Animated full color versions of the displays are available in Supplemental Materials. (B) Annotated, not-to-scale cartoon of the motion for different sections of the display in the edge-grouped and region-colors-similar (RCS) condition. Arrows within a region indicate relative direction of motion for dotted region texture. Arrows above or below a section indicate the relative motion direction of the vertical dividing edge for that section. The frequencies listed indicate the rate of oscillation associated with that display element (e.g. 1.0 Hz below a region arrow indicates a 1.0 Hz oscillation for texture dots in that region). “F” and “G” indicate anticipated figural or ground status (respectively) within the locally-biased section due to dot motion in relation to critical edge motion (C) Edge-grouped and region-colors-dissimilar (RCD) condition. (D) Edges-ungrouped and region-color-similar (RCS) condition. (E) Edges-ungrouped and region-color-dissimilar condition (RCD). Blue and green region colors within the locally-biased sections are replaced by dark gray and light gray here.

In Experiment 1 we tested both local and contextual influences on figure-ground organization within the same subjective report paradigm used in a recent study with normal individuals (Brooks & Driver, 2010). We presented LG (and control participants) with displays containing two edge segments. One edge segment had local cues to figure-ground organization (herein the *locally-biased edge*; Fig. 2A lower section, labeled 3). We expected that when control participants judged this section they would reliably report figure-ground organization consistent with the local cue. If LG has intact mechanisms for use of this local figure-ground cue, then he would do the same. Another edge segment (herein the *locally-ambiguous edge*; Fig. 2A upper section, labeled 2), in a different part of the scene, had no local cues to figure-ground organization. Previous work with normal observers has shown that figure-ground organization along this edge is influenced by the locally-biased edge. When judging figure-ground organization along the locally-ambiguous edge, control participants judged it as assigned in the same direction (e.g. to the left or the right) as the contextual edge, thereby showing a remote contextual influence (Brooks & Driver, 2010). Importantly, this contextual influence was evident only when the two edges were *grouped* by common motion and collinearity (Fig. 2B and C; see Supplementary Materials for animated examples). That is, the edges were aligned and moved in synchrony together. When the two edges were not grouped, due to different speed and phase of motion (Fig. 2D and E and Supplementary material animated examples), normal participants were equally likely to choose either region as figural along the locally-ambiguous edge.

If the remote contextual influence on figure-ground assignment is dissociable from local influences, and depends on normal functioning of intermediate visual areas, then we expected LG to show reduced or no contextual influence despite showing preserved effects of local figure-ground cues. LG and control participants made judgments of both the locally-biased edges and locally-ambiguous edges on separate trials interleaved within the same block. Judgments of locally-biased edges indexed effects of the local figure-ground cue whereas judgments of the locally-ambiguous section indexed contextual effects.

### 3.1 Methods

#### 3.1.1 Control participants

We tested 16 control participants (mean age = 24.6 years, 50% male, all right handed) with normal or corrected visual acuity. All participants gave informed consent and the procedures were approved by the UCL Research Ethics Committee.

#### 3.1.2 Displays and design

As in the Brooks and Driver (2010) study with control participants, each display comprised three sections (Fig. 2A, shown to scale); the top bipartite section, the bottom bipartite section, and the rectangular occluder between them. The top and bottom bipartite sections were each 5.3° square and were separated by a red rectangle (16.8° by 2.0°) centered at fixation. The vertical dividing edges within each bipartite section oscillated horizontally (0.85° oscillation distance) at either 1.0 Hz or 1.5 Hz. These vertical dividing edges (marked 2 and 3 in Fig. 2A) will henceforth be referred to as the *critical edges* because figure-ground organization along these edges was judged by the participants.

Either the top or the bottom bipartite section (equally often within each condition) contained a local cue to figure-ground organization. That is, it was *locally biased*. Edge-assignment for the locally-biased section's critical edge was determined by powerful dynamic cues to figural edge-assignment (Palmer & Brooks, 2008; Yonas, Craton, & Thompson, 1987). The critical edge moved synchronously with the sparse dot texture on one side of it, assigning the edge to that region. The dot texture on the other side moved in counter-phase to the critical edge, so that region became ground. In the locally-biased section, the dot-motion-determined figure was placed on the left or right side of the critical edge with equal probability. Likewise, the initial direction of motion for the critical edge of the locally-biased section was equally often leftward or rightward. This counterbalanced for the effects of the recently described figure-ground cue of advancing vs. receding motion (Barenholtz & Tarr, 2009). The other bipartite section within the display was *locally-ambiguous*. It contained no texture dots and had no other local cues to figure-ground assignment arising from its own critical edge and adjacent regions.

As in Brooks and Driver (2010), we manipulated the relationship between the locally-biased and locally-ambiguous sections of the display with (1) edge-grouping and (2) region-color-similarity and (3) measured perceived figure-ground organization in just one of these sections at a time (i.e. section judged was either the locally-biased or locally-ambiguous). Thus, the experimental design was a 2 × 2 × 2 within-subjects factorial design with these three factors. There were 16 repetitions for each condition within each block. Each participant completed two blocks total of 128 trials each.

(1) Edge-grouping factor: in edge-grouped conditions the two critical edges were collinear and oscillated together at the same speed (Fig. 2B and C). In edge-ungrouped conditions, the two edges oscillated at different frequencies and started out of phase (Fig. 2D and E). In edge-grouped conditions, the initial direction of motion for the edge of the locally-biased section was the same as for the locally-ambiguous section, whereas it began with opposite motion in edge-ungrouped conditions. Edge oscillation frequency in the locally-biased section was either 1.0 Hz or 1.5 Hz, with equal probability. In edge-grouped conditions, the frequency was the same for the locally-ambiguous section, whereas for edge-ungrouped conditions it was different.
(2) Region-color-similarity factor: In region-colors-similar conditions, the colors of the regions adjacent to the critical edge in both the locally-biased and locally-ambiguous sections were black (average 9.0 cd/m^2^, herein ‘black’) and white (average 53.5 cd/m^2^, herein ‘white’). In the region-colors-dissimilar conditions, locally-biased section regions were green (CIE *x* = 0.287, *y* = 0.592, 53 cd/m^2^) and blue (CIE *x* = 0.142, *y* = 0.069, 8 cd/m^2^) whereas the locally-ambiguous section's regions were black and white. White was made equiluminant to green and black was made equiluminant to blue. For control participants, the exact luminance for each color was determined for each participant individually by flicker photometry (Wagner & Boynton, 1972). This was done to ensure that the region colors were similar in luminance to the green and blue colors used in the region-color-dissimilar conditions. For LG, we used the control participants’ average luminance values for black and white. The color of the two regions within the locally-ambiguous section was counterbalanced (white on left or right), and the color in the locally-biased section was either the same (in region-color-similar conditions), or differed in being a green/blue combination (region-color-dissimilar conditions) with equal probability. In the latter case, contrast polarity across the edge (i.e. dark/light or light/dark) was counterbalanced by using either blue/green (on the left/right respectively) or green/blue instead.
(3) Section Judged factor: participants reported phenomenal figure-ground assignment for only one section of the display (upper or lower) in each block. Because the judged section was equally often locally-biased or locally-ambiguous, there were two separate sets of results. Judgments of the locally-biased section allowed us to measure whether the local figure-ground bias induced by the dots was indeed effective. Judgements of the locally-ambiguous section indicated whether figure-ground assignment of the locally-ambiguous edge was affected remotely by that of the locally-biased section, the critical issue in this study. The order (judge-top-section-first or judge-bottom-section-first) was counterbalanced across control participants. LG judged the bottom section in the first block and the top section in the second block.

#### 3.1.3 Procedure

For control participants, displays were presented on a 21-in. CRT computer monitor (60 Hz, 1280 × 1024 pixel resolution) in a dark, sound-attenuated testing booth. For LG, displays were presented on a 14-in. laptop LCD computer monitor (60 Hz, 1440 × 900 pixels resolution) in a darkened room at his home. The experimental procedure was controlled with Presentation software (Neurobehavioral Systems, Inc., http://www.neurobs.com). The viewing distance was approximately 60 cm for both LG and control participants. Each trial began with a central fixation cross for 1000 ms followed by the 2800 ms display. Participants reported, for just one section of the display (above the red bar in one block, below in the other block), which region appeared “in front”, using corresponding buttons (left or right mouse buttons). Participants were instructed to report their “first impression” as fast as possible and reaction times were recorded. For control participants, eye position was monitored throughout displays using an Eyelink 1000 eyetracker with a sampling rate of 250 Hz (SR Research Ltd., Kanata, Ontario, Canada: http://www.sr-research.com). Trials with >1° deviations from fixation or with pursuit eye movements (>0.5° regular oscillatory structure) during the displays were excluded (0.5% of all trials). For LG, due to practical limitations in his home environment, we were unable to formally monitor his fixation.

### 3.2 Results and discussion

#### 3.2.1 Statistics for comparing LG to control group

We assessed effects in control participants using a standard within-subject ANOVA and standard parametric *t*-test procedures. Unless otherwise specified, comparisons between LG and the control population were assessed using a modified *t*-test designed to compare single cases to control populations (Crawford, Garthwaite, & Howell, 2009; Crawford & Howell, 1998). We indicate this Crawford & Howell *t*-test as *t*_crawford_.

#### 3.2.2 Effectiveness of local cue; controls and LG show same effect

Within the locally-biased section (e.g. lower section of Fig. 2A), LG chose the locally-cue-indicated (i.e. the region consistent with the Palmer & Brooks dot-motion cue) region on 97.9% of trials (collapsed over edge-grouping and region-color-similarity conditions). This was not significantly different from the control participants (94.9%), *t*_crawford_(15) = 0.50, n.s. There were no significant differences between LG and controls in any of the locally-biased section's sub-conditions (all *p* > 0.31) nor did any of the sub-conditions (Table 1) differ from one another in controls (all *p* > 0.6). These results demonstrate LG's ability to use the local edge-region grouping cue (Palmer & Brooks, 2008; Yonas et al., 1987) to the same extent as controls to determine figure-ground organization locally within a region.

#### 3.2.3 Controls – contextual effect

Above we demonstrated that like control participants, LG can use the local cue to figure-ground organization within the locally-biased section as efficiently as control participants. Hence, we can now ask whether figure-ground organization within the locally-biased section (e.g. lower section of Fig. 2A) affected perception of the locally-ambiguous region remotely (e.g. upper section of Fig. 2A) as was shown for normally-developed participants in a previous study (Brooks & Driver, 2010), that is, whether LG showed contextual effects. We first verified that our control participants were, indeed, sensitive to the context when they were making figure-ground judgments regarding the locally-ambiguous region.

To quantify the contextual influence of the locally-biased section's figure-ground organization on that of the locally-ambiguous section, control participants’ responses for the locally-ambiguous section were coded according to whether they were *context-consistent*. For example, if the left side was figural (as indicated by the local cue) in the contextual locally-biased section, then a left response for the locally-ambiguous section was considered context-consistent. Context-consistency values significantly greater than 50% indicate a contextual influence in which the locally-ambiguous section acquires figure-ground organization in the same direction as the context. Context-consistency values equivalent to 50% indicate no contextual influence, i.e. an ambiguous figure-ground organization consistent with there being no local figure-ground cues within the locally-ambiguous section. Any context-consistency values significantly less than 50% would indicate a contextual influence but with figure assigned in the opposite direction from the context.

**Fig. 3:**
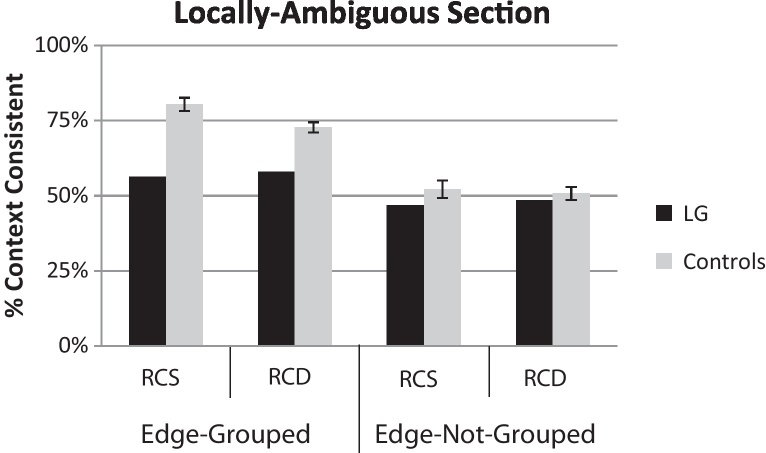
Locally-ambiguous section results from Experiment 1 for control participants (gray bars) and LG (black bars) as a function of edge-grouping and region-color-similarity (RCS, region-colors-similar conditions; RCD, region-colors-dissimilar condition). Scores represent the average percentage of trials on which the context-consistent region was chosen as figural. Values greater than 50% indicate a significant contextual effect on the locally-ambiguous section. Error bars represent standard error of the mean. Unlike controls, LG did not show a contextual influence in edge-grouped conditions.

When judging the locally-ambiguous section, control participants were significantly more likely to choose the context-consistent region as “in-front”/figural when edge-grouping was present than when the critical edges were ungrouped (Fig. 3, gray bars, both edge-grouped bars on left vs. both edge-not-grouped bars on right), *F*(1,15) = 123.1, *p* < 0.0001. In both edge-grouped conditions (stimuli: Fig. 2B and C; results: two gray bars on left in Fig. 3), control participants chose the context-consistent region significantly more often than 50%: region-colors similar (RCS), *t*(15) = 13.6, *p* < 0.0001; region-colors-dissimilar (RCD), *t*(15) = .13.4, *p* < 0.0001. In edge-not-grouped conditions (stimuli: Fig. 2D and E; results: two gray bars on right in Fig. 3), the context-consistent region was not chosen more often than 50%: region-colors-similar, *t*(15) = 0.72, *n.s.*; region-colors-dissimilar, *t*(15) = 0.34, *n.s.* This indicates that without edge-grouping, perception of the locally-ambiguous region was not affected by the context in normal participants. The strength of the contextual effect in edge-grouped conditions was significantly stronger in the region-colors-similar (RCS) condition than in the region-colors-dissimilar (RCD) condition (compare two left gray bars in Fig. 3), *F*(1,15) = 7.72, *p* < 0.01, but this had no effect in the edge-not-grouped conditions (i.e. an interaction between edge-grouping and region-color-similarity), *F*(1,15) = 5.40, *p* < 0.03. These perceptual reports of control participants for locally-ambiguous sections of the display, as a function of remote context, replicate previous work (Brooks & Driver, 2010) showing that figure-ground assignment propagates from the locally-biased section's critical edge to the locally-ambiguous critical edge but only when the two edges are grouped by collinearity and common motion.

#### 3.2.4 Pathological absence of contextual effect in LG

LG's data were scored with respect to context-consistency in the same manner as for control participants. LG chose the context-consistent side significantly less often than control participants in edge-grouped conditions (Fig. 3, edge-grouped gray bars vs. edge-grouped black bars): region-colors-similar (RCS), *t*_crawford_(15) = −2.62, *p* < 0.01; region-colors-dissimilar (RCD), *t*_crawford_(15) = −2.14, *p* < 0.04. For him, the percentage of context-consistent responses for the locally-ambiguous region was not significantly greater than 50% for any of the conditions (Fig. 3, all black bars vs. 50% using binomial test): edge-grouped/region-colors similar, *p*_binomial_ = 0.11; edge-grouped/region-colors-dissimilar, *p*_binomial_ = 0.11; edge-not-grouped/region-colors-similar, *p*_binomial_ = 0.13; edge-not-grouped/region-colors-dissimilar *p*_binomial_ = 0.10. For both the locally-biased section (*X*^2^ = 0.11, *p* < 0.99) and the locally-ambiguous section (*X*^2^ = 0.38, *p* < 0.99), the pattern of results did not differ between judging the top and bottom sections.

#### 3.2.5 Reaction times (RT)

LG's median RT (1629.4 ms) was significantly longer than the control participants’ average median RT (980.2 ms, SE = 50.4), *t*_crawford_(15) = 3.12, *p* < 0.007. Because we were more interested in the effects of the experimental factors on RT rather than overall speed, we normalized RTs by dividing each participant's RT in each condition by that participant's overall RT. For control participants, this normalized-RT was not significantly affected by edge-grouping, *F*(1,15) = 1.00, *p* < 0.33, or region-colors-similarity, *F*(1,15) = 0.11, *p* < 0.75. Normalized RT was marginally lower when judging locally-biased sections (1.03, SE = 0.06) than locally-ambiguous sections (0.97, SE = 0.06), *F*(1,15) = 3.71, *p* < 0.07. This indicates that they were marginally faster for locally-biased sections. There were no interactions of any of these factors (all *p* > 0.22). LG did not show any significant difference from the control population in any of the conditions for normalized RT (all *p*_crawford_ > 0.10).

#### 3.2.6 Experiment 1 summary

These results demonstrate that LG showed a pathological loss of contextual influence on figure-ground organization in the locally-ambiguous section of the display, despite showing intact effects of local figure-ground cues within the locally-biased section of the display. This is in contrast to control participants who showed a clear pattern of contextual influence in edge-grouped conditions, a pattern observed previously (Brooks & Driver, 2010).

## 4 Experiment 2 – no effect of context distance

In Experiment 1, the medial (closest to fixation) end of the locally-biased edge was separated from the medial end of the locally-ambiguous edge by 2° of visual angle. Although control participants showed substantial contextual influence (approximately 80% of the strength of the local cue, on average) at this distance, LG showed no significant influence of the context. In Experiment 2 we reduced the distance between the two edges to see whether LG would show any contextual influence at shorter distances between the locally-ambiguous edge and the source of contextual influence (i.e. the locally-biased edge). It is possible that the visual integration mechanisms that mediate contextual influence function in LG's brain but over smaller field of integration. Alternatively, LG may be unable to incorporate contextual influence into his figure-ground judgments even at a very short distance because his visual integration mechanisms are severely disturbed. In addition to testing the above issue, this experiment provides an opportunity to assess any effect of distance on control participants’ contextual influences, a factor not studied in previous work with this paradigm (Brooks & Driver, 2010).

### 4.1 Method

#### 4.1.1 Control participants

We tested 10 new control participants (mean age = 30.4 years, 60% male, 9 right handed, 1 left handed) with normal or corrected visual acuity. All participants gave informed consent and the procedures were approved by the UCL Research Ethics Committee.

#### 4.1.2 Displays and design

The displays were exactly the same as in Experiment 1 except that the thickness (i.e. vertical extent) of the red occluder bar was now either 2.0° (as in Experiment 1), or 1.0°, or 0.15°. When the occluder bar was thinner than in Experiment 1, the two bipartite sections remained the same size but were moved closer to fixation and remained adjacent to the (now thinner) occluder bar. The design was a 2 × 2 × 2 × 3 within-subjects factorial design with edge-grouping, region-color-similarity, section-judged (all preceding factors as in Experiment 1), and inter-edge distance as the factors. There were 16 repetitions of each condition resulting in 384 trials.

#### 4.1.3 Procedure

The procedure was the same as Experiment 1, except that Experiment 2 was longer and, therefore, split into 4 blocks by 3 self-limited breaks. Either the upper or lower section (counterbalanced across participants) was judged continuously for any given participant, resulting in judgments of the locally-ambiguous section on some trials and the locally-biased section on other trials (as in Experiment 1). LG participated in two separate (one month apart) sessions of 384 trials each. Due to experimenter error, he judged the top section in both sessions. This should not bias the results because in Experiment 1 no differences were observed for LG between top/bottom judgments (and likewise for control participants). On average, 2% of control participants’ trials were excluded due to eye movements (using the same thresholds as in Experiment 1).

### 4.2 Results and discussion

#### 4.2.1 Locally-biased section results

All data were scored as in Experiment 1. On average when judging the locally-biased section, control participants chose the cue-indicated region as figural on 97.6% of trials. This did not differ due to any of the experimental factors (see Table 2) or their interactions (all *p* > 0.1). LG chose the cue-indicated region on 99.5% of trials (collapsed across all conditions). This was not significantly different from that of control participants in any of the sub-conditions for locally-biased section judgments (all *p*_crawford_ > 0.25). These results replicate those of Experiment 1 and demonstrate that LG shows normal sensitivity to the local figure-ground cue within the locally-biased section.

#### 4.2.2 Contextual effect – control participants

Judgments of the locally-ambiguous section were scored as the percentage context-consistent as in Experiment 1. In judgments of the locally-ambiguous section, control participants chose the context-consistent region as figural significantly more often in edge-grouped conditions than in edge-not-grouped conditions, *F*(1,9) = 114.0, *p* < 0.0001 (two left gray bars vs. two right gray bars in Fig. 3A–C). In all edge-grouped conditions the context-consistent region was chosen as figural significantly more often than 50% indicating a significant contextual effect: 0.15°-Edge-grouped/RCS, *t*(9) = 16.65, *p* < 0.00001; 0.15°-Edge-grouped/RCD, *t*(9) = 7.58, *p* < 0.00001; 1.0°-Edge-grouped/RCS, *t*(9) = 11.75, *p* < 0.00001; 1.0°-Edge-grouped/RCD, *t*(9) = 8.07, *p* < 0.00001; 2.0°-Edge-grouped/RCS, *t*(9) = 11.62, *p* < 0.00001; 2.0°-Edge-grouped/RCD, *t*(9) = 4.63, *p* < 0.00001. Participant context-consistent responses were significantly greater than 50% in only one of the edge-not-grouped conditions when the occluder was thin (0.15°) and region colors were similar (RCS), *t*(9) = 3.63, *p* < 0.005. None of the other edge-not-grouped conditions (two right gray bars in Fig. 3A–C) were significantly greater than 50% (all *p* > 0.17). These results indicate that edge-grouping, when present, allowed the contextual influence from the locally-biased section to affect locally-ambiguous section judgments. This replicates the main result of Experiment 1 and the results of Brooks and Driver (2010).

There was also a main effect of region-color-similarity (RCS mean = 73.7%; RCD mean = 66.6%), *F*(1,9) = 8.74, *p* < 0.016, with region-color-similar trials showing more contextual influence. Unlike Experiment 1, though, there was no significant interaction of edge-grouping and region-color-similarity, *F*(1,9) = 0.006, *p* < 0.94. Instead, the three-way interaction of edge-grouping, region-color-similarity, and occluder thickness approached significance, *F*(2,18) = 3.05, *p* < 0.07, as we report for completeness. This three-way interaction might reflect a contextual influence mediated by region-color-similarity in the shortest inter-edge distance (0.15°) condition, even when edge-grouping was not present (i.e. the only edge-not-grouped condition to yield significantly greater than 50% context-consistent, see above). Context-consistent responses were not greater than 50% in edge-not-grouped conditions for other inter-edge distance values (see previous paragraph). There was no significant main effect of inter-edge distance, *F*(1,9) = 2.06, *p* < 0.15 indicating that controls were not significantly affected by the distance from the context over the range of distances that we tested. But the key question was whether or not contextual influences would emerge in LG when the biasing context was placed closer to the unbiased edge.

#### 4.2.3 No contextual effect for LG regardless of distance

**Fig. 4:**
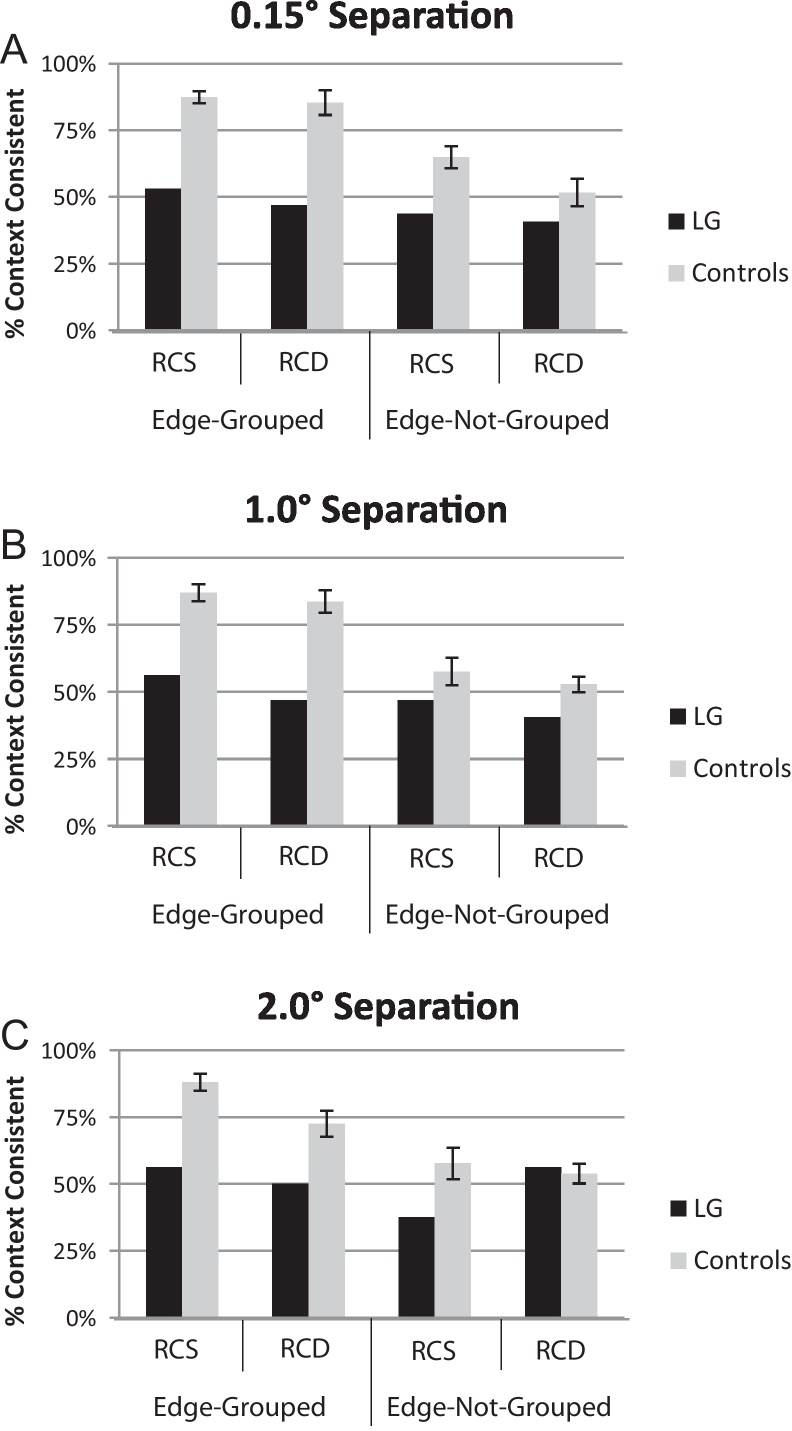
Locally-ambiguous section results from Experiment 2 for both control participants (gray bars) and LG (black bars) as a function of edge-grouping and region-color-similarity (RCS, region-colors-similar conditions; RCD, region-colors-dissimilar condition). Results are plotted separately for different separation distances between the display sections: (A) 0.15° separation. (B) 1.0° separation. (C) 2.0° separation. Scores represent the average percentage of trials on which the context-consistent region was chosen as figural. Values greater than 50% indicate a significant contextual effect on the locally-ambiguous section. Error bars represent standard error of the mean.

When judging the locally-ambiguous section, LG's context-consistent responses were not significantly different from 50% regardless of the distance between the context and the locally-ambiguous section (all *p*_binomial_ > 0.1; Fig. 4A–C black bars vs. 50%). Furthermore, LG showed significantly fewer context-consistent responses than control participants in all of the edge-grouped conditions (see Fig. 4A–C, two left gray bars vs. two left black bars; inferential statistics in Table 3) except in the 2.0°-edges-grouped/RCD condition. The reason for this exception is not clear. There were no significant differences between LG and control participants in any of the edges-ungrouped conditions (Fig. 4A–C, two right black bars vs. two right gray bars; all *p* > 0.11), where no contextual effects were expected.

## 5 Experiments 1 and 2 summary and discussion

In sum, despite showing preserved influence of *local* cues on figure-ground assignment, LG did not incorporate *contextual* information into his figure-ground judgments of the locally-ambiguous section, even with very short distances between the locally-biased and locally-ambiguous edges. In contrast, control participants showed consistent contextual influences in edge-grouped conditions at all of the distances that we tested. Importantly, LG's lack of a contextual effect cannot be attributed to general inability to perform figure-ground judgments, because he performed equivalently to control participants on this task when the figure-ground cue was local to the judged edge. Furthermore, trials measuring the local and contextual influences were interleaved randomly within each block which makes strategic effects unlikely. The overall information on the screen was the same on trials that reflected contextual influence and those that reflected local influence. The only difference was whether the section judged was that with the local figure-ground cue (locally-biased) or the locally-ambiguous section. Our results show that despite reporting normal perceived depth/figural assignment in response to local cues along an edge, LG shows no contextual influence from nearby edges. This dissociation between local and contextual figure-ground influences suggests that they depend, at least partially, on independent mechanisms.

One possible explanation of LG's impaired visual integration derives from previously observed functional abnormalities in LG's brain. Although activity in response to visual stimulation is relatively normal in LG's primary visual cortex and some higher-order cortical areas, intermediate visual areas (e.g. V2/V3) show deactivation in response to visual stimulation compared to baseline (Gilaie-Dotan et al., 2009). Thus, it is possible that contextual influences on figure-ground depend critically on normal functioning of these intermediate visual areas. In contrast, local figure-ground cues can be used by LG suggesting that this can be computed without need for normal functioning of V2/V3. One specific possibility is that local figure-ground cues are computed in a bottom-up manner in early visual cortex, whereas contextual influences depend more on feedback from intermediate and higher visual areas with larger receptive fields. The importance of feedback in contextual figure-ground effects is supported by both neurophysiological work (Heinen, Jolij, & Lamme, 2005; Sugihara et al., 2011) and computational modeling (Craft et al., 2007; Kienker et al., 1986; Sajda & Finkel, 1995). For instance, the onset time of contextual figure-ground influences on border ownership signals in V2 neurons does not depend critically on the distance of the contextual cues (Sugihara et al., 2011). It is difficult to account for these results in a model using only horizontal interactions within the same cortical area. Our results are consistent with the notion that contextual influences on figure-ground organization, at least in the form studied here, may require intermediate visual areas.

## 6 Experiment 3: preserved function of weaker local cues

In Experiments 1 and 2, we found that LG showed normal figure-ground organization within the locally-biased section of the displays. However, when judging the locally-ambiguous section using the same task, LG, in contrast to the control participants, was not influenced by the locally-biased *contextual* section. This was true even when the context was at very short distances. We have interpreted this as reflecting a dissociation between local and contextual mechanisms of figure-ground assignment. That is, our data suggest that different and functionally independent neural circuits implement local and contextual influences on figure-ground organization.

The local figure-ground cue within locally-biased sections in Experiments 1 and 2 was quite strong, causing both control participants and LG to see the predicted side as figural approximately 95–98% of the time. In contrast, the context-consistent side within the locally-ambiguous region was chosen as figural on only 75–80% of trials by control participants. This means that the contextual effect is a significantly weaker figure-ground cue than the local cue, *t*(15) = 6.40, *p* < 0.000001, comparing local-predicted to ambiguous context-consistent region choice. Only this weaker effect was absent in LG. On conceivable alternative interpretation of the results in Experiment 1 and 2 is therefore that only weaker figure-ground cues (such as the contextual cue) are selectively disrupted in LG. In this account, there would be no need to explain the results with a dissociation between local and contextual figure-ground mechanisms. Instead, a single damaged mechanism may show a deficit when operating with impoverished or non-optimal input (i.e. a weaker figure-ground cue in our case), but it can operate normally when the input is optimal (i.e. a strong figure-ground cue in our case). If that is the case with LG's figure-ground processes, then an effect of cue strength cannot be taken as evidence for a dissociation.

To investigate this alternative account, we tested LG and control participants on several local figure-ground cues that varied in strength from 50% to 93% choice of the predicted region (for controls). Equivalent performance of LG and control participants across this range of local cues, would demonstrate that cue strength does not account for LG's lack of a contextual figure-ground effect and would support our case for a dissociation between local and contextual mechanisms of figural assignment.

**Fig. 5:**
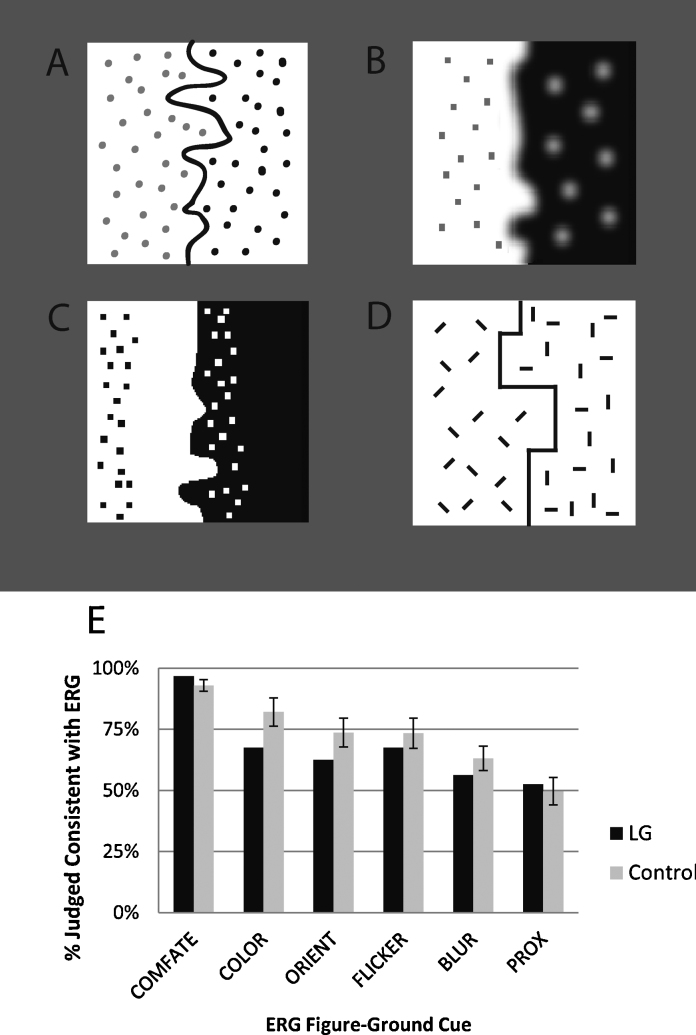
Stimuli and results of Experiment 3. (A–D) Example stimuli with four edge-region grouping (ERG) factors of differing strength. In all of these examples, ERG predicts assignment of the edge to the right side. Additional factors such as color of the region, which side was ERG, were counterbalanced. See the text for details. (A) Color similarity between edge and right region texture elements. (B) Blur similarity. (C) Proximity of texture elements and edge. (D) Orientation similarity between edge components and region texture elements. (E) Results of Experiment 3 showing percentage of trials on which LG (black bars) and control participants (gray bars) chose the ERG-predicted region as figural. This is shown for 6 different ERG factors. The ERG factors are ordered, from left to right, with decreasing strength for control participants. Error bars on control participant data represent standard error of the mean.

For these additional tests, we chose local figure-ground cues that were variations on the edge-region grouping (ERG) proposal (Palmer & Brooks, 2008). This includes the cue used in the locally-biased sections of Experiments 1 and 2. The ERG proposal holds that an edge will be assigned to the adjacent region with which it best *groups*. For instance, the vertical edge within the locally-biased sections of Experiment 1 and 2 oscillated back-and-forth. The dot texture in one of the adjacent regions also oscillated with the same speed and phase. In terms of perceptual grouping, this region was perceptually grouped with the edge by the classic Gestalt grouping cue of common fate. According to the ERG hypothesis, this grouped region would be figurally assigned to the edge. The other adjacent region oscillated at a different speed and thus was not grouped by common fate. Therefore, it should be perceived as background. As shown in the locally-biased section results of Experiments 1 and 2, ERG via common fate grouping caused robust figural assignment with 90–100% choice of the predicted region as figural. Other grouping cues can also be used to associate the edge to an adjacent region. For instance, the texture elements within one adjacent region may be grouped with the edge by color similarity (Fig. 5A), blur similarity (Fig. 5B), proximity (Fig. 5C), orientation similarity (Fig. 5D), or flicker synchrony (i.e. the elements onset and offset synchronously with the edge; see the animated Supplementary Fig. S5). These ERG grouping principles tend to produce weaker figural assignment than common fate, ranging from approximately 50–80% in control participants. Hence, using this range of cues provides a good basis for testing the alternative cue strength account of our results. If LG chooses the ERG-predicted region as often as controls even for weaker ERG cues, then this rules out the cue strength account.

### 6.1 Method

#### 6.1.1 Control participants

The control participants were 11 university students who participated for course credit. These were different participants than in Experiments 1 and 2. There were 6 males and 5 females. The average age was 20.4 years. All participants gave informed consent and the procedures were approved by the UCL Research Ethics Committee.

#### 6.1.2 Displays and design

The display on each trial comprised a 5.3° square bipartite stimulus at the center of the screen. This bipartite stimulus was equivalent in size to the locally-biased section of Experiments 1 and 2. The bipartite stimulus was divided in half vertically by a pseudorandom curvy edge. The algorithm for generating this edge is describe in detail in Brooks and Driver (2010). This algorithm ensured that the regions were balanced for convexity and size. The edge was either a contrast edge (Fig. 5B and C) or a line edge (Fig. 5A and D) depending on the particular ERG grouping cue on that trial.

There were six different ERG cue types: common fate (60 trials), flicker synchrony (20 trials), color similarity (40 trials), blur similarity (40 trials), proximity (20 trials), and orientation similarity (20 trials). This resulted in a total of 200 trials which were presented in a random order in a within-subjects design. The number of trials was different for each ERG cue because each ERG cue varied different features of the display and thus required different counterbalancing. These details are described separately for each ERG cue below. Control participants completed one experimental session. LG completed two sessions on the same day and the results of these two sessions were combined (400 total trials). Each trial had a different curvy edge between the regions but edges were the same across the two sessions for LG.

It was common to all ERG cues that the side predicted to be figural by ERG (left or right) was counterbalanced within the trials for each ERG cue. Other features differed for each ERG grouping cue as follows.

Common Fate: There were three versions of common fate grouping. There were 20 trials of each version and each produced strong figural assignment in a previous study but did vary slightly in strength (Palmer & Brooks, 2008). (A) One matched the locally-biased section in Experiments 1 and 2 exactly, that is, the edge moved with the texture in one region and the texture dots in the other region moved out of phase and at a different speed. The speeds and other factors were the same. (B) Another version involved a static edge. The edge grouped with static dots in one region while dots in the other region moved. (C) A third version involved a moving edge grouped with moving dots in one region. The dots in the other region were static and thus that region was background. All three versions were counterbalanced for the color of the ERG-predicted side (black or white) and the starting direction of motion of the edge. The frequency of oscillation of the edge was randomly assigned on each trial from the two frequencies used in Experiments 1 and 2.

Flicker Synchrony (Supplemental Fig. S5): there were two versions of flicker synchrony with 10 trials of each. (A) In one type the edge flickered in synchrony with and thus was grouped with the dots on one side of the edge. The dots in the other region did not flicker and thus did not group with the edge. ERG predicts assignment of the edge to the grouped, flickering side. (B) In the other version, the edge was not flickering and thus grouped with the region containing stable, non-flickering dots. The dots in the other region were flickering. ERG predicts figural assignment of the edge to the stable, non-flickering region in this case. In both conditions the flickering items flickered at 7.5HZ.

Color similarity (Fig. 5A, grayscale version): there were two versions of ERG by color similarity grouping with 20 trials each. (A) In one type, the edge was a colored line (either red or green, equiluminant by photometer). The texture dots in one region shared the same color as the edge. The texture dots in the other region were a different color. ERG predicts assignment of the edge to the region with the dots of the same color as the edge. (B) The other version was the same as above except that the entire region (aside from the dots) was filled with a lower contrast version of the same color. This provides a basis for color similarity grouping between the edge, the texture elements, and the rest of the region. ERG predicts assignment of the edge to the region filled with the same hue and colored dots. Both of these versions were counterbalanced for the color (red or green) of the ERG predicted side.

Blur similarity (Fig. 5B): the edge was either sharp or blurred with a using a Gaussian kernel with a 6-pixel radius (0.18°). The texture dots in one region were sharp. The texture dots in the other region were blurred with the same parameters. The peak contrast of the blurred dots was kept constant to that of the sharp dots. ERG predicts that the edge will be assigned to the region with the same degree of blur as the edge. The trials were counterbalanced for the color and degree of blur of the ERG predicted side.

Proximity (Fig. 5C): the texture dots within one region were all within 1° of the contrast edge (i.e. grouped by proximity) whereas the texture dots in the other region were distal from the curvy edge and within 1° of the outer edge of the region. ERG predicts assignment of the edge to the region with the dots proximal to the edge. The trials were counterbalanced for the color (black or white) of the ERG predicted region.

Orientation similarity (Fig. 5D): the edge was a line edge. The edge was composed of either horizontal and vertical line segments or diagonal line segments. One region was filled with horizontal and vertical line segments. The other was filled with diagonal line segments. ERG predicts assignment of the edge to the region with texture elements of similar orientation. Trials were balanced for whether the ERG predicted side contained horizontal/vertical or diagonal line segments.

#### 6.1.3 Procedure

The display apparatus and settings were identical to those in Experiments 1 and 2 except that no eyetracking was available for this experiment. Before the experiment began, the participants were shown a faces-vase ambiguous stimulus to explain the concept of figure-ground organization and they were also shown one example of each stimulus (which was not included in later testing) to demonstrate the various types of stimuli to expect. On each trial, the stimulus was presented for a limited amount of time and participants were asked to indicate the direction of figural assignment across the edge (left or right) as fast as possible, giving their first impression. The stimulus duration for common fate displays was 1500 ms and for flicker synchrony displays it was 792 ms (6 cycles). Displays for the other ERG cues were presented for 400 ms. There was a 1000 ms ITI.

### 6.2 Results and discussion

#### 6.2.1 Results comparing LG to controls for six ERG cues

The response on each trial was coded as either consistent or inconsistent with the ERG prediction (i.e. which side was predicted figural by ERG). The results were collapsed across all of the counterbalancing factors and subtypes for each ERG cue to produce six values per participant, representing the percent ERG consistent responses for each of the six different ERG cues. This was done separately for LG's two runs through the paradigm. The results from those two runs were averaged.

Control participants’ average percentage choice of the ERG predicted region ranged from 50 to 92% across the 6 ERG cues tested (Fig. 5E, gray bars). LG's performance (Fig. 5E, black bars) did not different significantly from that of control participants for any of these cues (Crawford & Howell test results in Table 4). Furthermore, LG's performance across the cues correlated significantly with the pattern of results for control participants, *r* = 0.885, *p* < 0.01. For every ERG cue except proximity, LG selected the ERG predicted region significantly more often than 50% (by binomial test): common fate, *p* < 0.00001; flicker synchrony, *p* < 0.02; color similarity, *p* < 0.0006; blur similarity, *p* < 0.04; and orientation similarity, *p* < 0.03. Control participants showed the same pattern. They were significantly different from 50% for all of the ERG cues except proximity: common fate, *t*(10) = 17.96, *p* < 0.00001; flicker synchrony, *t*(10) = 3.79, *p* < 0.004; color similarity, *t*(10) = 5.56, *p* < 0.00001; blur similarity, *t*(10) = 2.61, *p* < 0.02; and orientation similarity, *t*(10) = 4.01, *p* < 0.002. For proximity, LG did not choose the ERG predicted region significantly more often than 50%, *p* < 0.11. This was in agreement with control participants who also did not differ from 50% for proximity, *t*(10) = −0.064, *p* < 0.95.

#### 6.2.2 Implications of results

The results presented above show that LG performed equivalently to control participants for six local ERG cues varying in strength from 50 to 93% choice of the predicted region. Furthermore, for five of the six cues, LG selected the ERG predicted region significantly more often than 50% demonstrating an influence of the local ERG cues. This was in contrast to LG's performance in the locally-ambiguous section of Experiments 1 and 2. There, LG never chose the context consistent region on significantly more than 50% of trials, and he consistently showed performance different from that of controls. For the proximity ERG cue, both LG and control participants were not above 50%, again showing equivalent performance.

LG's normal sensitivity to local ERG figure-ground cues of varying strength rules out the cue strength interpretation of LG's contextual impairment. LG's pattern of results is therefore most likely represents a selective impairment in integrating contextual information into figure-ground assignment. Given his intact performance on a range of local figure-ground cues, this provides clear evidence for a dissociation between local and contextual figure-ground mechanisms. This has implications for computational and neurobiological models of figure-ground organization. It may constrain the structure of those models to implement local and contextual figure-ground processes in a functionally dissociable manner.

## 7 Experiment 4: familiarity and convexity influences on figure-ground organization

**Fig. 6:**
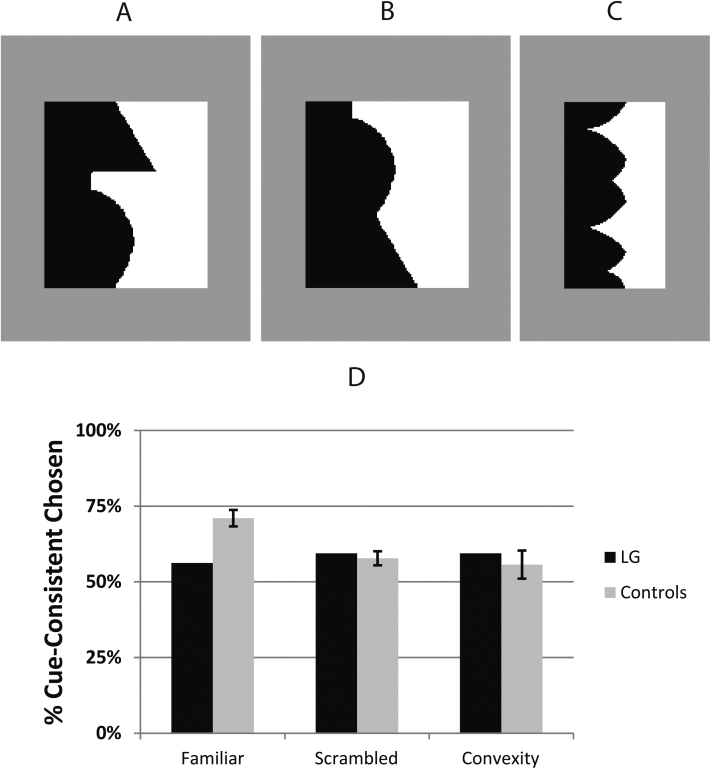
Stimuli and results of Experiment 4. Stimuli were presented briefly for 100 ms. (A) Familiar bipartite stimulus depicting a silhouette of a lamp along the left side of the edge. (B) Scrambled stimulus created by breaking lamp edge (stimulus shown in panel A) at deep concavities and rearranging parts. (C) Convex bipartite stimulus with the convex region to the left. (D) Experiment 4 results as the percentage of trials on which the cue-consistent region was chosen. Black bars represent LG's score. Gray bars represent the average for control participants. Error bars represent standard error of the mean.

In this experiment we tested whether LG shows any influence of object familiarity on figure-ground organization. Typically developed individuals tend to assign figure to the side of an edge that depicts a familiar, common object. For instance, the edge in Fig. 6A depicts the silhouette of a lamp along the left side of its border and participants are more likely to see this region as figural compared to the other region (e.g. Peterson & Gibson, 1991, 1994a). However, typically developed participants are less likely to choose that same side of the edge when the familiar shape is broken into components and these are rearranged to form a *scrambled* edge (Fig. 6B) with similar parts and low-level features but no overall familiarity (Peterson, Gelder, Gerhardstein, & Bachoud-levi, 2000; Peterson, Gerhardstein, Mennemeier, & Rapcsak, 1998). The difference between figure-ground assignment in these two cases represents the effect of shape familiarity on figure-ground organization.

**Fig. 7:**
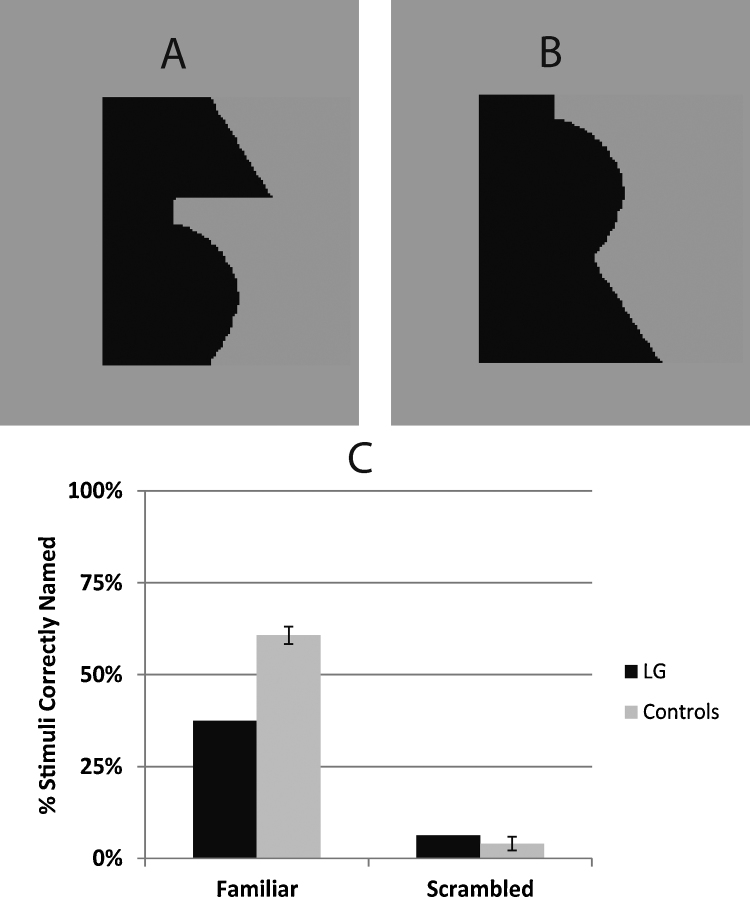
Stimuli and results of the conscious naming part of Experiment 4. (A) Familiar lamp stimulus. (B) Scrambled lamp stimulus. (C) Results showing the percentage of familiar and scrambled stimuli that could be correctly named by LG and control participants with unlimited exposure duration. Black bars represent LG's score. Gray bars represent the average for control participants. Error bars represent standard error of the mean.

The mechanisms of familiarity influences on figure-ground organization have sparked debate. Some have suggested that object knowledge functions in a top-down, interactive manner (Vecera & O’Reilly, 1998, 2000) perhaps relying on feedback connections from shape-sensitive higher-order visual areas to border-ownership neurons in lower-order areas. Others have suggested that these influences may arise through early (106–156 ms: Trujillo, Allen, Schnyer, & Peterson, 2010) or bottom-up processing of familiarity cues (Peterson, 1999) and are dissociable from conscious object recognition (Peterson et al., 2000). Given LG's profoundly pathological function in intermediate visual areas and impaired object recognition, testing him provides an opportunity to assess the role of intermediate visual areas in familiarity influences on figure-ground organization. Accordingly, we showed him (and control participants) familiar and scrambled bipartite stimuli like those in Fig. 6A and B, adapted from previous work on figure-ground familiarity influences with normal observers (Peterson & Gibson, 1994a; Peterson et al., 2000; Peterson & Kim, 2001). We asked participants to indicate which side (left or right) was figural/in-front. In line with previous work, we expected controls to choose the side depicting the common object more often in the intact than the scrambled condition. If LG shows a similar difference between these two conditions, it would show preserved familiarity influences on figure-ground organization despite his object recognition difficulties and suggest that intermediate visual cortex has no necessary role in familiarity effects on figure-ground. To assess his explicit object recognition, subsequent to the figure-ground test we gave him an explicit naming task with the same exact edges used in the figure-ground task. However, in this explicit naming task, the edges did not appear in bipartite stimuli. Instead, each edge appeared as the edge of a single black region on a fully surrounding gray background (see Fig. 7). The explicit naming task also gave participants unlimited exposure time unlike the figure-ground task which had only a brief exposure (100 ms) in order to encourage first impression responses.

We also took advantage of this opportunity to test one further known local cue to figure-ground organization, which is convexity. Normally-developed participants can show a slight tendency to choose convex regions (left region in Fig. 6C) as figural (Kanizsa & Gerbino, 1976; Peterson & Salvagio, 2008; Stevens & Brookes, 1988) compared to concave regions. This may arise because convexity is correlated with real depth across edges in natural scene statistics (Burge, Fowlkes, & Banks, 2010). The convexity effect is quite weak, however, in simple bipartite displays like those used here (Peterson & Salvagio, 2008), and as it turned out it was not significant here even for control participants. As a consequence, the main purpose of the convex displays became simply to provide a further control for whether any effect of familiarity vs. scrambled might be due to inadvertent convexity differences between those stimuli. If convexity alone explains figural biases toward the familiar and scrambled regions, then the figural/scrambled scores should be no greater than the score for the bipartite convexity stimulus that was specifically designed to contain a significant convexity imbalance.

### 7.1 Method

#### 7.1.1 Control participants

We tested 16 control participants (mean age = 27.2, 10 males, 14 right-handed) with normal or corrected visual acuity. These participants were different from those in Experiments 1–3. All participants gave informed consent and the procedures were approved by the UCL Research Ethics Committee.

#### 7.1.2 Displays and design

The display equipment was the same as the previous experiments. The background of the screen was a neutral gray color (33 cd/m^2^). Each stimulus comprised a centrally located bipartite stimulus with one white (68 cd/m^2^) region and one black (0 cd/m^2^) region which shared a vertically oriented *critical edge*. As in previous work (e.g. Peterson & Gibson, 1994b), the bipartite stimulus was 3° (visual angle) in height and 2.5–4.2° in width. Three different types of critical edges were used: familiar, scrambled, and convex. There were 32 bipartite stimuli of each of these types with a total of 96 different edges.

The critical edge of each familiar stimulus was the silhouette of a familiar object on one side of the edge (e.g. Fig. 6A). For instance, the left side of the critical edge in Fig. 6A depicts a lamp. 75% of the familiar stimuli were taken from previous studies by Peterson and colleagues (Gibson & Peterson, 1994; Peterson et al., 1998, 2000; Peterson & Kim, 2001). See Supplementary Materials (Table S1 and Fig. S7) for download links and details of which stimuli from each set were used. We supplemented these stimuli with 8 from our own set and other stimuli by Peterson (shown in Supplementary Materials Fig. S6) in order to increase the number of repetitions in each condition. The two regions of the bipartite familiar stimuli were approximately equal in area.

The scrambled stimuli were formed by manipulating the familiar images described above. Familiar edges were broken into segments at the prominent concave cusps and the segments randomly rearranged (Peterson et al., 1998, 2000). This resulted in an image sharing the same edge segments/parts as the original familiar image but without the familiarity of the overall configuration (e.g. Fig. 6B). Because scrambled objects share the same edge-segments (and thus many low-level image features such as convexity) with the familiar objects, they serve as good control stimuli (for comparison to familiar stimuli) to isolate effects of overall familiar shape. Familiar stimuli from the Peterson sets had scrambled counterparts in those sets. Stimuli from our own set were modified according to the same rules described above.

The convex stimuli (e.g. Fig. 6C) were obtained from previous research on the effect of convexity (Peterson & Salvagio, 2008) on figure-ground organization. See the Supplementary Materials for a list of which stimuli were used from this set. The method for constructing the stimuli is described in the Peterson and Salvagio (2008) paper (their Experiment 1 stimuli and apparatus) and this followed previous work (Stevens & Brookes, 1988).

The familiar, scrambled and convex regions, herein referred to as the *cue-consistent region*, were counterbalanced for color (black or white) and side (left or right) within each type. Hence, participants saw an equal number of white cue-consistent region regions on the left as black cue-consistent region regions on the left and the same balance of colors on the right. The assignment of colors and sides to particular stimuli was randomly determined on each run of the program and thus different for each participant.

#### 7.1.3 Procedure

Display equipment was the same as in Experiments 1 and 2. Each control participant and LG viewed the same exact set of 96 stimuli (32 each for familiar, scrambled and convex) twice, once in each of two blocks. The order of the stimuli within each block was random and different between the two blocks. Each trial started with a 1000 ms fixation cross followed by the bipartite stimulus presented for 100 ms. The control participants were instructed to indicate which side of the bipartite stimulus, left or right, appeared to be “in front”/figural using corresponding mouse buttons. Eye movements were monitored for control participants and trials were excluded for deviations from fixation greater than 1° (1.3% of trials on average). LG was instructed to maintain fixation as in the previous experiments.

#### 7.1.4 Explicit object recognition – naming test

This test was conducted on a separate day, approximately 3 months after the figure-ground procedure described above. LG and 7 new control participants (5 males, 2 females; all right-handed) saw a single black rectangle with one articulated vertical edge (on left side half of trials) and 3 straight edges. The articulated edge either depicted one of the familiar objects (Fig. 7A) or one of the scrambled objects (Fig. 7B) from the Experiment 4 figure-ground task. The stimulus appeared after a 1000 ms fixation period. The participant was instructed to press a mouse button as soon as they recognized the shape depicted along the articulated border so that reaction time could be estimated. The stimulus disappeared after this response. Then the participant immediately verbally named the stimulus. If they could not recognize it, they could either guess or pass without answering. The next trial began after the experimenter recorded the verbal response (several seconds).

### 7.2 Results and discussion

The figure-ground responses were coded as the percentage of trials on which the cue-consistent region was chosen as figural. For instance, if the left region was familiar, scrambled, or convex and the participant chose the left region, this was counted as a cue-consistent response. Values significantly greater than 50% indicate an effect of the cue that biased figural assignment toward the cue-consistent side. Values that are not different from 50% indicate no significant effect of the cue.

#### 7.2.1 Control participants figure-ground results

Control participants chose the cue-consistent region as figural significantly more often than 50% in both the familiar, *t*(15) = 7.74, *p* < 0.0001, and scrambled conditions, *t*(15) = 3.36, *p* < 0.004 (Fig. 6D, gray bars). The cue-consistent region was chosen significantly more often in the familiar condition than in the scrambled condition, *t*(15) = 6.47, *p* < 0.0001. These results demonstrate that the familiar edge configurations caused figure-ground organization to be assigned in that direction. This is consistent with previous results by others (e.g. Peterson & Gibson, 1991, 1994a).

Convex regions were not chosen significantly more often than 50% of the time by control participants (55.7%), *t*(15) = 1.21, *n.s*. This initially appears inconsistent with previous studies which demonstrated that convex regions are more likely to be judged figural than concave regions (Kanizsa & Gerbino, 1976; Stevens & Brookes, 1988). However, those studies primarily used multi-partite displays with multiple alternating convex and concave regions. The convexity effect is relatively weak in bipartite displays like those used here (Peterson & Salvagio, 2008). Peterson and Salvagio observed only 57% choice of the convex region for bipartite displays and with a slightly larger sample size than our control group this was significantly different than chance. They found that convexity effects are more pronounced in displays with multiple alternating concave and convex regions, which were not used here.

#### 7.2.2 Figure-ground results – LG

Unlike control participants who reliably chose the familiar and scrambled regions as figural, LG's cue-consistent choices were not significantly greater than 50% for the familiar (*p*_binomial_ < 0.10), scrambled (*p*_binomial_ < 0.08), or convex stimuli (*p*_binomial_ < 0.08). LG chose the cue-consistent region 14.8% less often than the average control participant in the familiar condition. But to isolate the impact of familiarity, the critical measure is the difference between familiar and scrambled conditions. Control participants were 14.1% (SEM = 1.91%) more likely to select the cue-consistent region in familiar stimuli than in scrambled stimuli. For LG, this difference was −3.1%, significantly different from control participants, *t*_crawford_(15) = −2.19, *p* < 0.04.

#### 7.2.3 Explicit naming results

To assess naming accuracy, each object was given a basic-level category label by the experimenter in advance of the experiment. The verbal responses were judged as either correct or incorrect (based on this label) by the first author and independently by a colleague blind to the hypotheses and participant identities. Ratings of the two judges were averaged. Agreement of these two sets of scores was 97%. LG was significantly impaired with the familiar objects both in naming accuracy (Fig. 7C, *t*_crawford_(6) = −3.49, *p* < 0.007) and in RT (LG: 11,689 ms vs. controls: 3742 ms, *t*_crawford_(6) = 3.093, *p* < 0.02). In contrast, he was not different from controls in the Scrambled condition (accuracy: Fig. 7C, *t*_crawford_(6) = 0.41, *p* < 0.34), where naming was difficult even for normal observers, although he was very slow: RT (LG: 15,941 ms vs. controls: 6734 ms, *t*_crawford_(6) = 1.989, *p* = 0.065).

Although LG was significantly impaired at explicit object naming, he was nevertheless able to recognize a small proportion of the stimuli. We analyzed the results of the figure-ground task separately for those familiar stimuli that he could explicitly name and those that he could not name. We found that even for familiar stimuli that were explicitly recognizable he chose the cue-consistent region only 54% of the time. For unrecognized familiar stimuli he chose the cue-consistent region 64% of the time. This indicates that even when explicit recognition is intact, LG is unable to use this information to affect figure-ground assignment.

#### 7.2.4 Experiment 4 summary and discussion

In line with his previously observed object recognition difficulties, LG was significantly impaired in explicitly naming familiar stimuli. More importantly, the results of Experiment 4 showed a significant impairment of LG's ability to use familiarity cues in figure-ground organization even for the small number of stimuli that he was able to explicitly name. In contrast, control participants successfully named almost twice as many stimuli as he did and consistently used familiarity to determine their figure-ground assignment. This outcome suggests that intermediate visual areas may play a critical role in mediating familiarity influences on figure-ground organization. Our results also suggest that familiarity influences can dissociate from local image-based figure-ground mechanisms that were shown to be intact in Experiments 1 and 2.

An important and classic issue in perceptual organization is whether different processes occur relatively early or late (e.g. Palmer et al., 2003; Palmer & Nelson, 2000; Palmer & Rock, 1994) in time or higher vs. lower in the anatomical hierarchy of visual cortical areas. For instance, does figure-ground organization occur before object recognition or after (cf., Peterson, 1999; Peterson & Gibson, 1994a)? As described earlier, electrophysiological evidence suggests that familiarity influences on figure-ground processing can arise relatively early in time (Trujillo et al., 2010) and without explicit object recognition (Peterson et al., 2000). From our current work, the relative timing of familiarity influences and the processing of local figure-ground cues is not completely clear. We can conclude that local figure-ground cues do not depend strictly on the same mechanisms as familiarity influences because familiarity can be impaired even when local cues function normally. It is possible that this is because local figure-ground cues are computed by functionally independent neural circuits before the circuits that compute familiarity. However, this is not necessarily the case. One alternative is that familiarity is independently computed in a parallel pathway simultaneously with, or even earlier than, local figure-ground cues. This architecture could show a dissociation between the two mechanisms even though they operate simultaneously. Assuming that LG is available for future electrophysiological testing, we may be able to address this issue more clearly in follow-up work. Although the data that we have cannot currently resolve the more difficult issue of relative timing of familiarity and local figure-ground influences, the pattern of dissociations that we have observed does indicate that mechanisms which compute at least some local figure-ground cues (such as the ERG cues used here) are dissociable from those that compute familiarity influences on figure-ground organization.

## 8 Summary and general discussion

We tested figure-ground assignment performance by a rare developmental agnosic who has object recognition problems and profoundly deactivated intermediate visual areas in response to visual stimulation, despite apparently normal brain structure. As shown previously with fMRI (Gilaie-Dotan et al., 2009), visual responses of his primary visual cortex are relatively normal and higher order visual cortical areas also show positive activation to visual stimulation (although with reduced category selectivity), while retinotopic areas V2/V3 respond abnormally. This unusual case provides an opportunity to test the roles that these intermediate visual areas V2 and V3 may normally play in local, contextual, and familiarity influences on figure-ground assignment. We found that LG was significantly less sensitive to contextual influences on figure-ground organization than control participants. This was true even for very short distances between the figure and contextual inducer. Importantly, his sensitivity to local figure-ground influence was unimpaired. This suggests that the neural mechanisms responsible for computing figure-ground organization/border ownership based on local information along a contour are functionally dissociable from those mechanisms that incorporate contextual information. This dissociation may, for instance, arise from the use of different grouping circuits for local and contextual figure-ground influences with selective impairment of the contextual mechanism in LG. This is broadly consistent with the proposal that perceptual grouping operates at various different levels throughout visual processing rather than being a single unitary mechanism (Palmer et al., 2003). To further test this hypothesis we encourage further work with rare cases such as LG to test other grouping influences in addition to the ones we examined here. LG also failed to show an influence of familiarity on figure-ground organization. This demonstrates that familiarity influences are mediated by different mechanisms than those based on computing the other local image-based figure-ground cues used in our experiments (edge-region grouping local cue in Experiment 1 and part-familiarity and convexity in Experiment 2).

Previous work with LG has suggested problems with visual integration as the primary dysfunction of the visual perception system. For instance, in a recent study on facial emotion perception, LG was unable to integrate contextual visual information from the rest of the body to inform facial emotion perception (Aviezer et al., 2011). The findings of the current study suggest that LG's contextual integration difficulties may be pervasive, occurring at both lower and higher levels of the perceptual system.

As LG exhibits robust deactivation (compared to baseline) of intermediate visual areas V2 and V3 in response to visual stimulation, it is clear that local figure-ground mechanisms do not depend on the normal function of these areas because local figure-ground influences were normal in LG. In contrast, it is likely that contextual and familiarity influences on figure-ground organization do depend critically on these areas for normal functioning. Future work on typically developed participants may use transcranial magnetic stimulation or other techniques that disrupt cortical function to test the causal relationship between these brain areas and contextual and familiarity influences on figure-ground organization. Our work also constrains the architecture of computational models of figure-ground organization. These models should contain dissociable mechanisms for local and contextual figure-ground computations in order to be consistent with our observations.

## Figures and Tables

**Table 1:** Experiment 1 results for locally-biased section judgments.

	Edges-grouped RCS	Edges-grouped RCD	Edges-not-grouped RCS	Edges-not-grouped RCD
Control participants	95.3% (SE = 1.4%)	94.7% (SE = 1.7%)	95.8% (SE = 1.6%)	93.9% (SE = 1.5%)
LG	96.9%	96.9%	100.0%	98.4%

*Note*: Values represent the percentage of trials on which the locally-cue-indicated region was chosen as figural as a function of edge-grouping and region-color-similarity. RCS, region-colors-similar conditions. RCD, region-colors-different conditions. Scores are presented separately for LG and control participants. Control participant values in parentheses represent standard errors of the mean.

**Table 2:** Experiment 2 results for locally-biased section judgments.

		Edges-grouped RCS	Edges-grouped RCD	Edges-not-grouped RCS	Edges-not-grouped RCD
0.15° Separation	Control participants	96.9% (SE = 1.1%)	98.7% (SE = 0.8%)	98.1% (SE = 1.3%)	97.5% (SE = 1.4%)
	LG	100%	100%	100%	100%
1.0° Separation	Control participants	98.1% (SE = 1.4%)	100% (SE = 0%)	96.9% (SE = 1.9%)	98.1% (SE = 0.9%)
	LG	100%	100%	100%	100%
2.0° Separation	Control participants	96.2% (SE = 1.4%)	98.1% (SE = 0.9%)	96.2% (SE = 1.4%)	96.2% (SE = 1.0%)
	LG	100%	100%	100%	96.9%

**Table 3:** Crawford & Howell *t*- and *p*-values comparing LG and control participants for locally-ambiguous section judgments in experiment 2.

	Edges-grouped RCS	Edges-grouped RCD	Edges-not-grouped RCS	Edges-not-grouped RCD
0.15° Separation	*t*_crawford_(9) = −4.60	*t*_crawford_(9) = −2.48	*t*_crawford_(9) = −1.56	*t*_crawford_(9) = −0.64
	*p* < 0.0009	*p* < 0.02	*p* < 0.11	*p* < 0.31
1.0° Separation	*t*_crawford_(9) = −2.94	*t*_crawford_(9) = −2.66	*t*_crawford_(9) = 0.63	*t*_crawford_(9) = −1.25
	*p* < 0.01	*p* < 0.02	*p* < 0.31	*p* < 0.17
2.0° Separation	*t*_crawford_(9) = −2.92	*t*_crawford_(9) = −1.40	*t*_crawford_(9) = −1.01	*t*_crawford_(9) = 0.20
	*p* < 0.01	*p* < 0.14	*p* < 0.22	*p* < 0.38

**Table 4:** Crawford & Howell *t*- and *p*-values comparing LG and control participants for six ERG cues used in Experiment 3.

ERG cue	Crawford & Howell *t*-value	*p*-Value
Common fate	*t*_crawford_(10) = 0.456	0.347
Flicker synchrony	*t*_crawford_(10) = −0.274	0.373
Color similarity	*t*_crawford_(10) = −0.727	0.293
Blur similarity	*t*_crawford_(10) = −0.392	0.357
Proximity	*t*_crawford_(10) = 0.147	0.384
Orientation similarity	*t*_crawford_(10) = −0.547	0.330
