# Supplementary figures and images for: Preserved local but disrupted contextual figure-ground influences in an individual with abnormal function of intermediate visual areas
Source: Neuropsychologia. 2012 Jun;50(7):1393–407. doi: 10.1016/j.neuropsychologia.2012.02.024 (PMC3405515; doi:10.1016/j.neuropsychologia.2012.02.024)

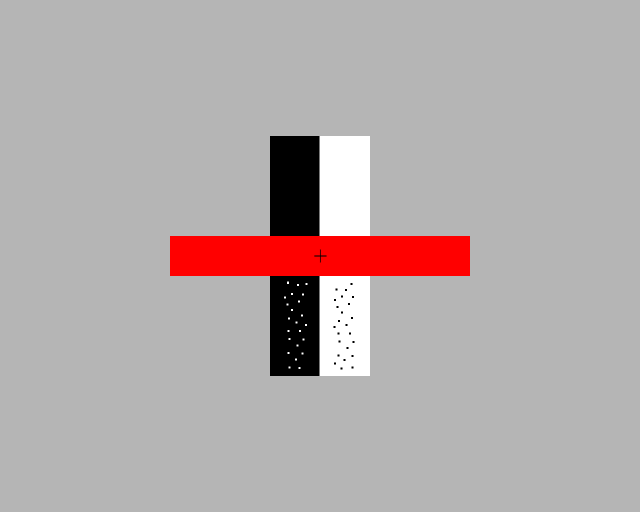

Supplement: Supplementary file 1 [file mmc1.gif]

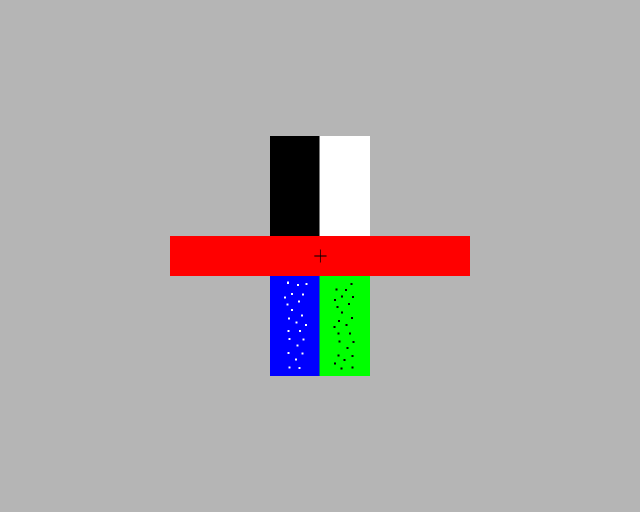

Supplement: Supplementary file 2 [file mmc2.gif]

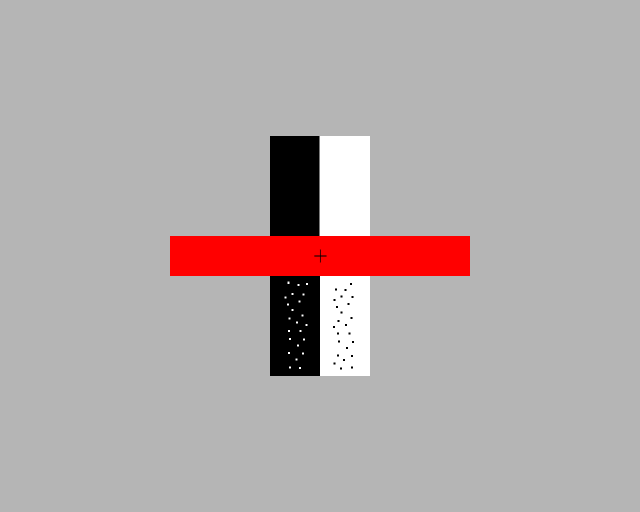

Supplement: Supplementary file 3 [file mmc3.gif]

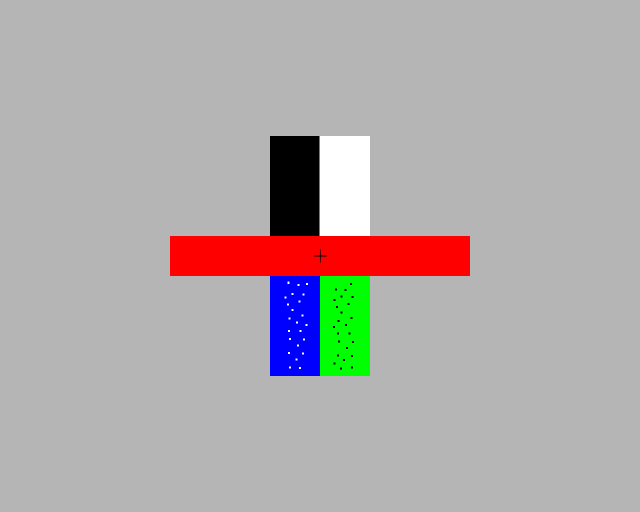

Supplement: Supplementary file 4 [file mmc4.gif]

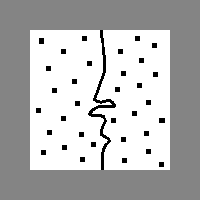

Supplement: Supplementary file 5 [file mmc5.gif]
