# Supplementary material for: Preserved local but disrupted contextual figure-ground influences in an individual with abnormal function of intermediate visual areas
Source: Neuropsychologia. 2012 Jun;50(7):1393–407. doi: 10.1016/j.neuropsychologia.2012.02.024 (PMC3405515; doi:10.1016/j.neuropsychologia.2012.02.024)
Supplement: Supplementary file 6 [file mmc6.pdf]

SUPPLEMENTARY MATERIALS

Preserved local but disrupted contextual figure-ground influences in an individual with abnormal function of intermediate visual areas

Joseph L. Brooks, Sharon Gilaie-Dotan, Geraint Rees, Shlomo Bentin, Jon Driver

EXPERIMENT 1 & 2 STIMULI

Figure S1 – S4 (animated GIFs supported separately)

These are animated versions of Figure 2B-E. The files are animated GIF format. The animation can be seen by opening the file in a web browser or animated GIF viewer.

Each display comprised a red horizontally oriented bar with superimposed fixation cross and one bipartite section above and one bipartite section below it. Each bipartite section contained a vertically oriented dividing edge (luminance contrast) which we call the critical edge. In this example, the locally-ambiguous section is located above the red bar and the lower section is the locally-biased section in this case. The location of the locally-ambiguous and locally-biased sections was counterbalanced in Experiment 1. For all examples S1-S4, the edge within the locally-biased section (lower section here) is biased toward the left region (i.e. left region figure). Both control participants and LG judged the figure-ground assignment in the locally-biased section to be consistent with this local figure-ground cue on over 90% of trials. This shows normal function of local figure-ground cues for LG (see Results and Discussion Experiments 1 & 2)

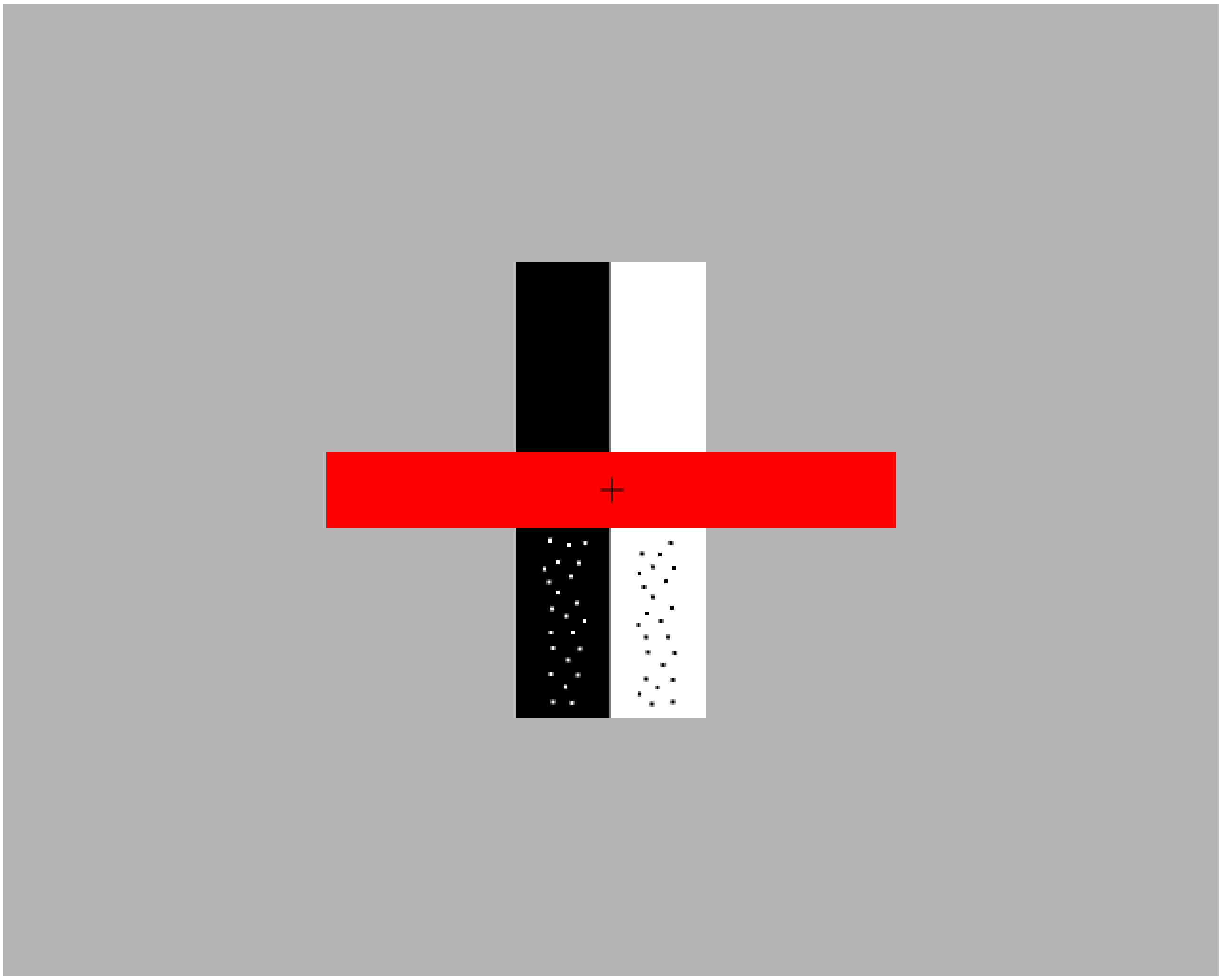

See animated Supplemental file FigureS1.gif (open file in web browser or animated GIF viewer) or

http://www.icn.ucl.ac.uk/brooks/LG/

Figure S1. Animated example of Figure 2B. The lower (locally-biased) and upper (locally-ambiguous) sections are grouped by both edge-grouping and region color similarity. Edge grouping reflects the common motion of the edges in the two sections. Color similarity reflects the matching colors of the two sections. For control participants, in this condition the locally-ambiguous section's figure-ground organization tended to be influenced by that of the locally-biased section. Specifically, if the locally-biased edge was assigned leftward (by the local cue) then control participants tended to perceive the locally-ambiguous section's edge as assigned leftward (about 75% of the time). LG, on the other hand

showed no such contextual influence of one section on the other. Within the locally-ambiguous section, he was equally likely to choose the left and right sides for the locally-ambiguous section.

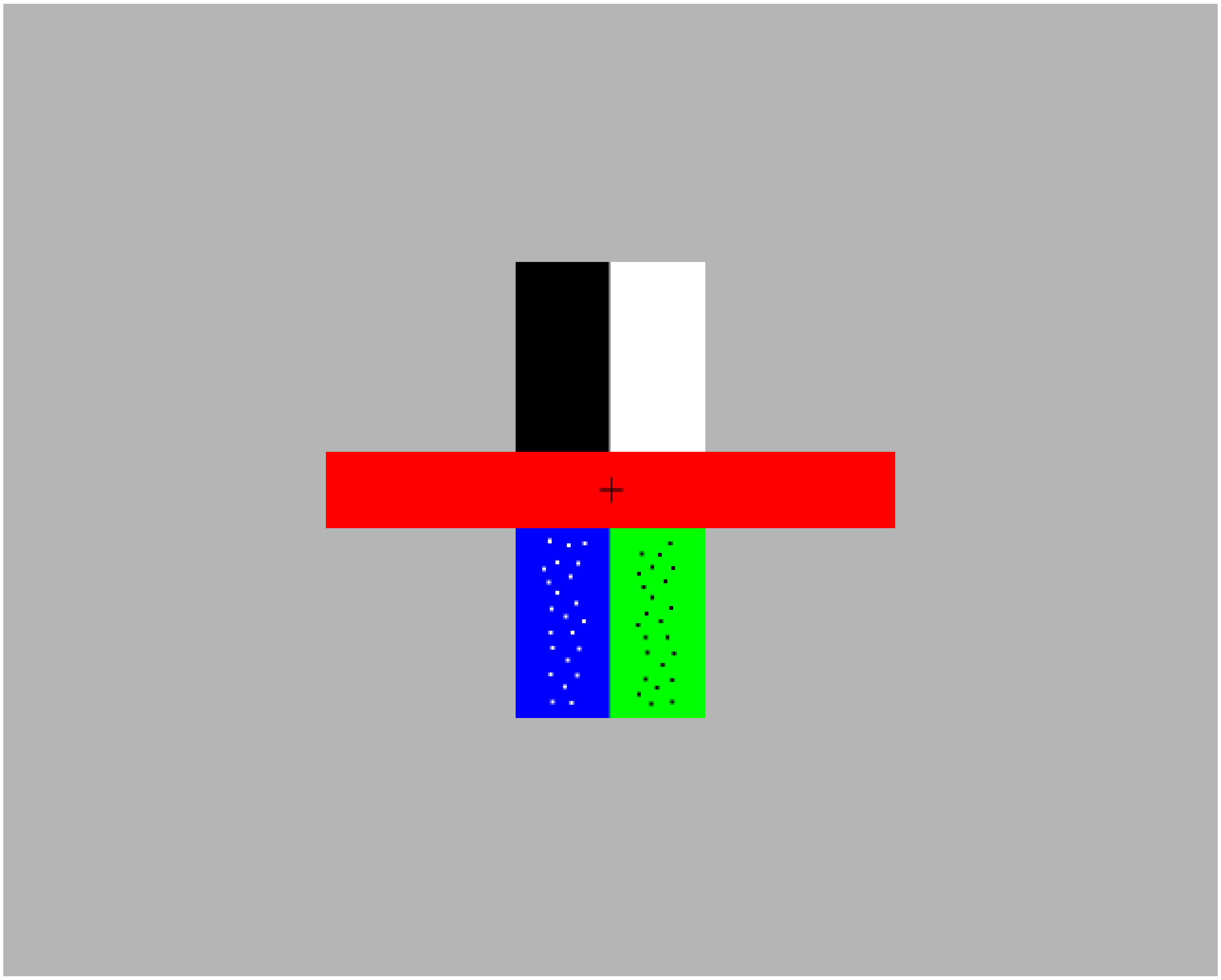

See animated Supplemental file FigureS2.gif (open file in web browser or animated GIF viewer) or http://www.icn.ucl.ac.uk/brooks/LG/

Figure S2. Animated example of Figure 2C. The lower (locally-biased) and upper (locally-ambiguous) sections are grouped by edge-grouping only. The colors of the two sections differ which makes them ungrouped by color dissimilarity. In this condition, control participants tend to report that figure-ground

organization of the locally-ambiguous section tends to be influenced by that of the locally-biased section. However, this effect is slightly weaker than when both edge-grouping and color similarity are present (Figure S1). LG shows no contextual effect in this condition, perceiving the edge as assigned leftward and rightward equally often regardless of edge assignment in the contextual (locally-biased) section.

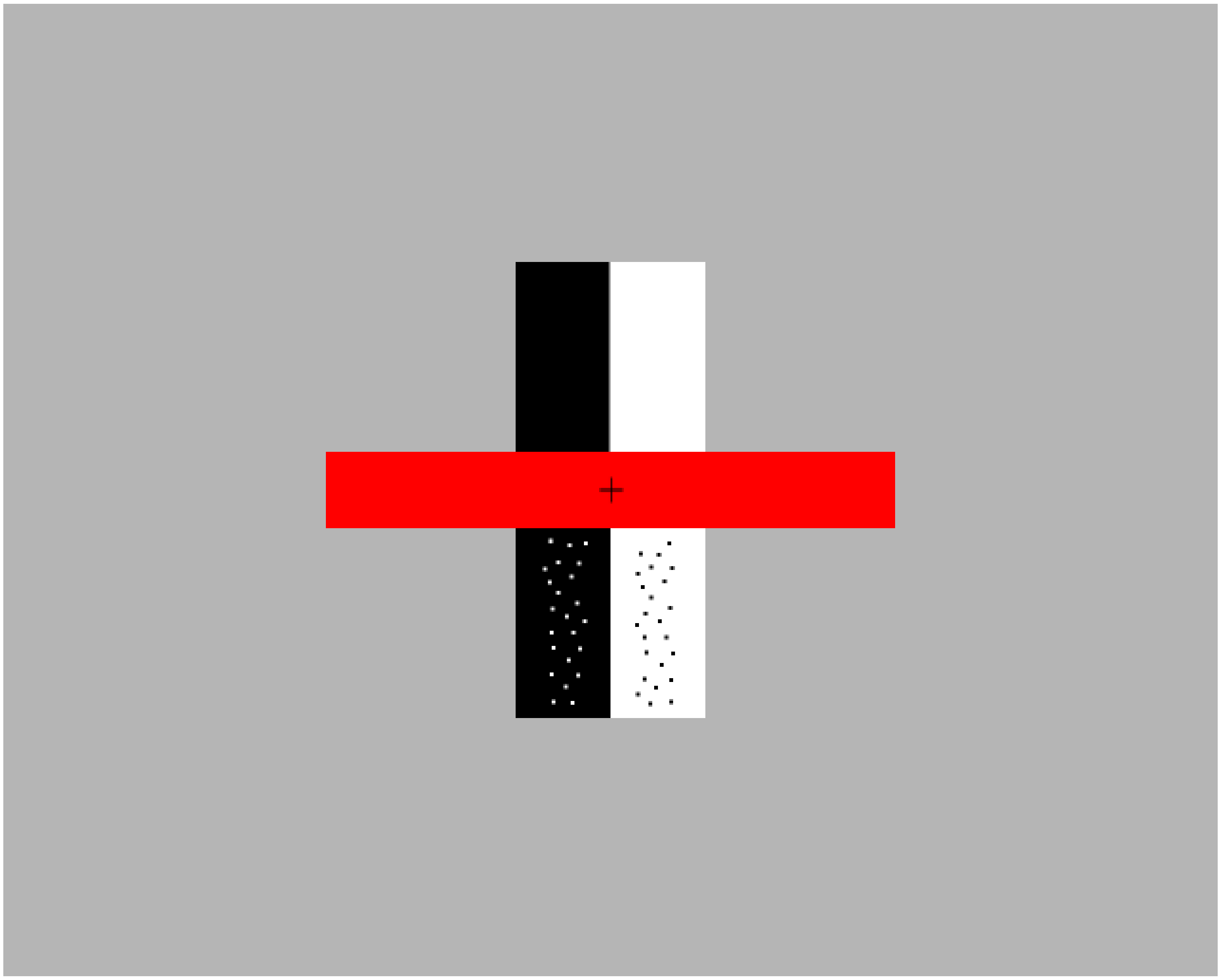

See animated Supplemental file FigureS3.gif (open file in web browser or animated GIF viewer) or http://www.icn.ucl.ac.uk/brooks/LG/

Figure S3. Animated example of Figure 2D. The lower (locally-biased) and upper (locally-ambiguous) sections are grouped by color-region-similarity only. The sections are not grouped by edge-grouping because the edges in the two sections move at different speeds. In this condition, for BOTH LG and control participants, the locally-ambiguous section's figure-ground organization is NOT influenced by that of the locally-biased section. Thus, the locally-ambiguous section is judged to be ambiguous (i.e. edge equally often assigned left and right).

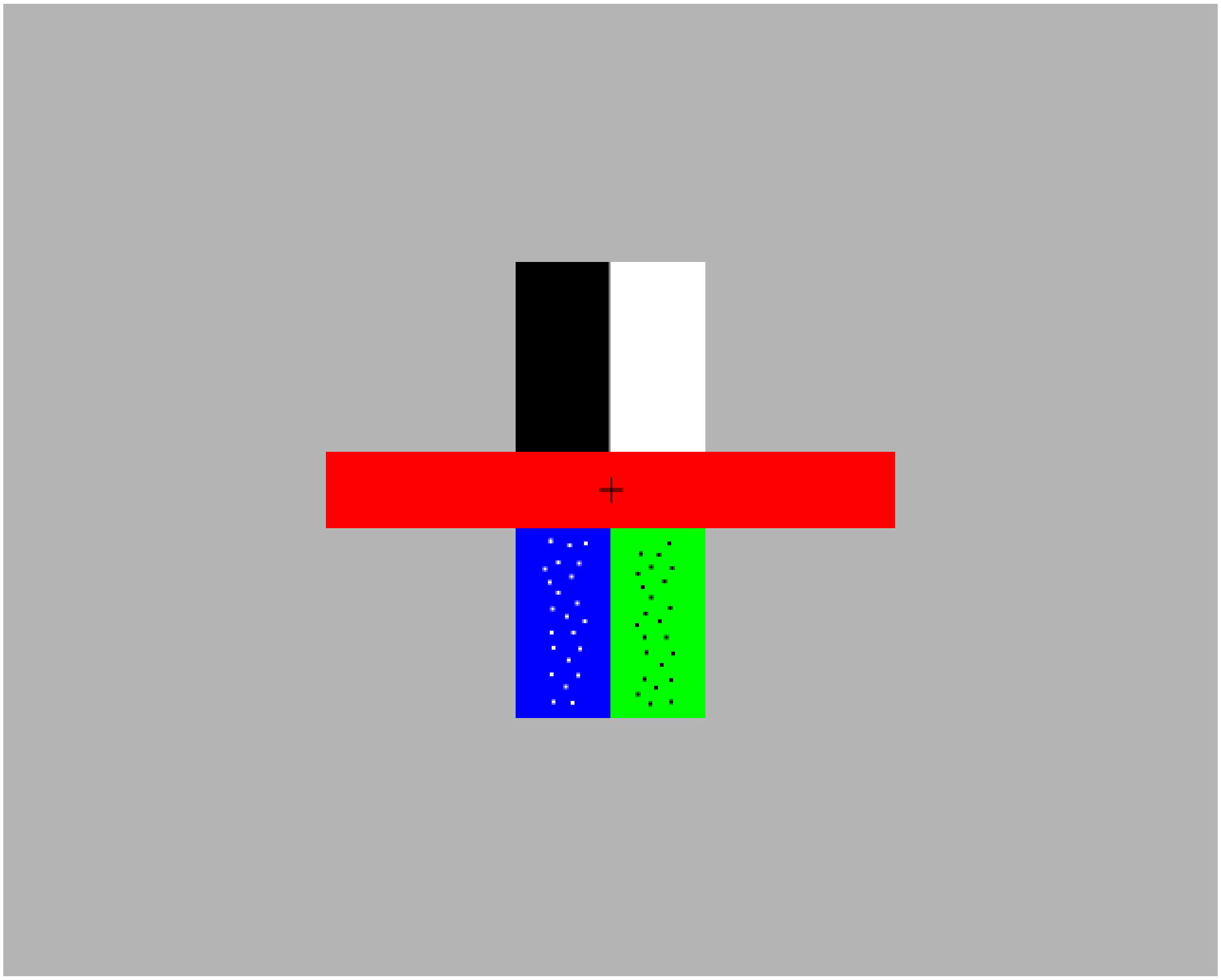

See animated Supplemental file FigureS4.gif (open file in web browser or animated GIF viewer) or

http://www.icn.ucl.ac.uk/brooks/LG/

Figure S4. Animated example of Figure 2E. The lower (locally-biased) and upper (locally-ambiguous) sections are NOT grouped by either edge-grouping or region color similarity. In this condition, for BOTH LG and control participants, the locally-ambiguous section's figure-ground organization is NOT influenced by that of the locally-biased section. Thus, the locally-ambiguous section is judged to be ambiguous (i.e. edge equally often assigned left and right).

EXPERIMENT 3 STIMULI

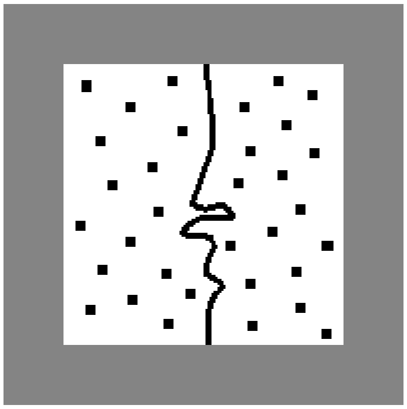

See animated Supplemental file FigureS5.gif (open file in web browser or animated GIF viewer) or http://www.icn.ucl.ac.uk/brooks/LG/

Figure S5. An animated example of edge-region grouping (ERG: Palmer & Brooks, 2008) by flicker synchrony in a bipartite display as used in Experiment 3. The edge flickers in synchrony with (and thus groups with) the texture dots in one of the regions. The texture dots in the other region flicker out of phase. The grouped region is usually assigned to be figure for control participants. In this example, figural assignment is to the right side based on flicker-ERG.

EXPERIMENT 4 STIMULI

75% of the familiar and scrambled stimuli were selected from those used by Peterson and colleagues (Peterson & Kim, 2001; Peterson et al., 2000; Peterson, Gerhardstein, Mennemeier, & Rapcsak, 1998). They came from two sets. Both sets can be accessed at http://www.u.arizona.edu/~mapeters/Stimuli . Table S1 details which stimuli of each set were used in Experiment 4. The side of the familiar/scrambled shape and the contrast polarity across the edge were counterbalanced in the experiment, i.e. participants saw an equal number of white familiar/scrambled regions on the left as black familiar/scrambled regions on the left and the same balance of colors on the right. The assignment of colors and sides to particular stimuli was randomly determined on each run of the program and thus different for each participant. The additional 25% of the familiar and scrambled stimuli were created in-house or obtained from other sources. The familiar versions of these are shown in Figure S6. The Peterson and Kim pictures had to be modified to become bipartite stimuli. The modified versions are shown in Figure S7.

OMEFA - http://www.u.arizona.edu/~mapeters/Stimuli/OMEFA/OMEFA.zip

Peterson & Kim- http://www.u.arizona.edu/~mapeters/Stimuli/PetersonKim2001/PetersonKimStimuli.zip

EXPERIMENT 4 CONVEX STIMULI

All of the convex stimuli were obtained from the Peterson and Salvagio set downloaded from http://www.u.arizona.edu/~mapeters/Stimuli/Convexity%20Context%20Stimuli.zip . The filenames of pictures that we used from this set are shown in Table S2. The side of the convex shape and the contrast polarity across the edge were counterbalanced in the experiment, i.e. participants saw an equal number of white convex regions on the left as black convex regions on the left and the same balance of colors on the right. The assignment of colors and sides to particular stimuli was randomly determined on each run of the program and thus different for each participant.

Table S1.

Names of Experiment 4 stimuli from Peterson and colleagues sets

Object Depicted	Stimulus Set
Apple	OMEFA
bell	OMEFA
coffee pot/kettle	OMEFA
Face	OMEFA
fire hydrant	OMEFA
Girl	OMEFA
guitar	OMEFA
Ice cream cone	OMEFA
lamp	OMEFA
light bulb	OMEFA
pear	OMEFA
pine tree	OMEFA
pineapple	OMEFA
seahorse	OMEFA
snowman	OMEFA
Stop	OMEFA
Tree	OMEFA
umbrella	OMEFA
wine glass	OMEFA
wrench	OMEFA
Bird	Peterson&Kim
duck	Peterson&Kim
flower	Peterson&Kim
teddy bear	Peterson&Kim

Table S2

List of filenames from Peterson & Salvagio set used in Experiment 4 convexity condition

Filenames			
cvx1101.bmp	cvx1110.bmp	cvx1206.bmp	cvx1302.bmp
cvx1103.bmp	cvx1111.bmp	cvx1207.bmp	cvx1303.bmp
cvx1104.bmp	cvx1112.bmp	cvx1208.bmp	cvx1304.bmp
cvx1105.bmp	cvx1201.bmp	cvx1209.bmp	cvx1305.bmp
cvx1106.bmp	cvx1202.bmp	cvx1210.bmp	cvx1306.bmp
cvx1107.bmp	cvx1203.bmp	cvx1211.bmp	cvx1307.bmp
cvx1108.bmp	cvx1204.bmp	cvx1212.bmp	cvx1308.bmp
cvx1109.bmp	cvx1205.bmp	cvx1301.bmp	cvx1309.bmp

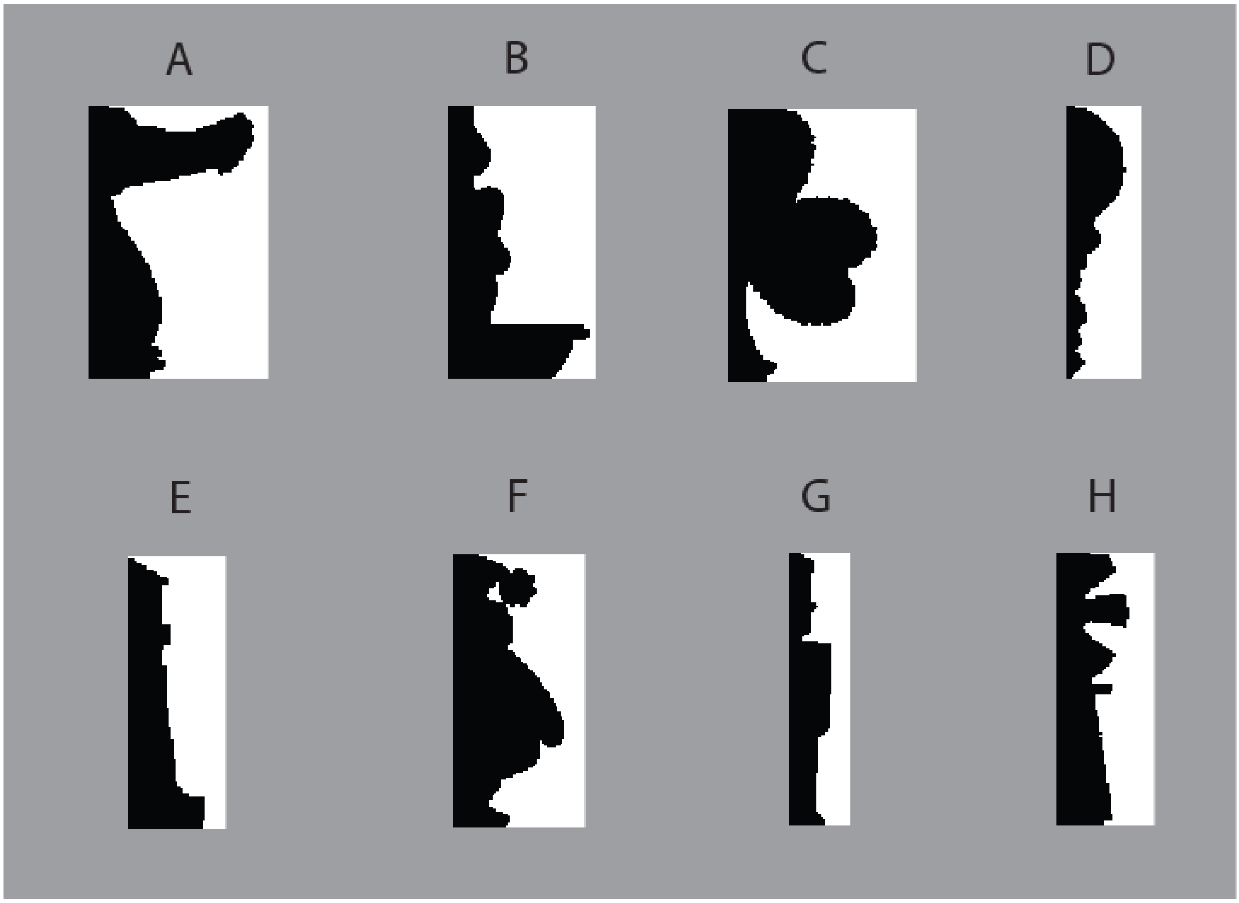

Figure S6. Experiment 4 familiar bipartite stimuli used in addition to those obtained from Peterson sets available on their website. For all of the examples here, the familiar object is depicted along the left side of the edge. The side of the familiar shape and the contrast polarity across the edge were counterbalanced in the experiment. (A) Alligator, (B) candle, (C) clover, (D) key, (E) lighthouse, (F) Santa Claus, (G) soldier, (H) windmill.

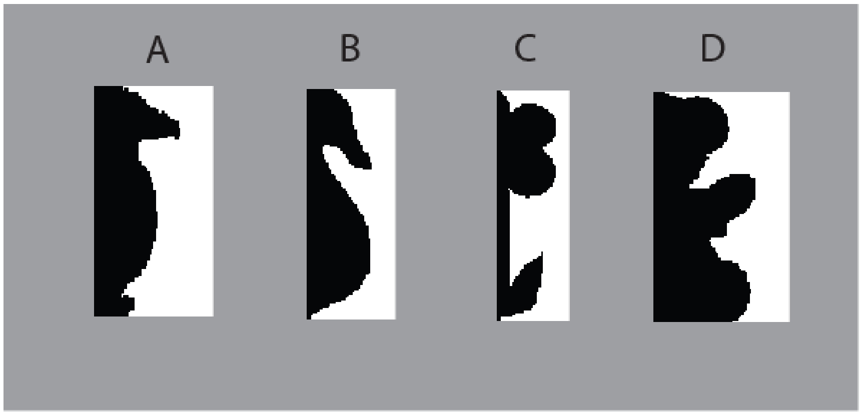

Figure S7. Experiment 4 familiar bipartite stimuli modified from the Peterson & Kim set. For all of the examples here, the familiar object is depicted along the left side of the edge. The side of the familiar shape and the contrast polarity across the edge were counterbalanced in the experiment. (A) Bird, (B) Duck, (C) Flower, (D) Teddy Bear.
